# PROTOCOL: Employee work motivation, effort, and performance under a merit pay system: A systematic review

**DOI:** 10.1002/cl2.70001

**Published:** 2024-10-30

**Authors:** Cédric Velghe, Anders McIlquham‐Schmidt, Pinar Celik, Martin Storme, Stan De Spiegelaere

**Affiliations:** ^1^ The VIGOR Unit Ghent Belgium; ^2^ CAIDEMA Hellerup Denmark; ^3^ Université Libre de Bruxelles Brussels Belgium; ^4^ IESEG School of Management Lille France; ^5^ Ghent University Ghent Belgium

**Keywords:** compensation, merit pay, pay‐for‐performance, work effort, work motivation, work performance

## Abstract

This is the protocol for a Campbell systematic review. The objectives are as follows: One goal of this systematic review is to identify whether merit pay predicts employee work motivation, effort, and performance; a second goal is to determine whether the association between merit pay and subsequent employee work motivation, effort, and performance is stronger depending on the actual relationship between the performance ratings and merit increases received, as well as on the perceived relationship by employees between their performance and their pay; a third goal is to identify whether the association between merit pay and subsequent employee motivation depends on what type of motivation is measured (i.e., intrinsic vs. extrinsic/general work motivation).

## 1 BACKGROUND

### 1.1 The problem, condition, or issue

For organizations to survive and thrive, their employees must perform well. An important issue for organizations to solve on this front is how to enhance and maintain employee work motivation and effort, as they are essential determinants of employee work performance (Van Iddekinge et al., 2018; Van Iddekinge et al., 2023). A widely used practice aimed at enhancing employee motivation and effort is merit pay, a member of the family of pay‐for‐performance (P4P) practices, which includes all forms of compensation where how much employees are paid depends to a certain extent on their performance (Heneman & Werner, 2004; Rynes et al., 2005). Other examples of P4P are profit sharing, sales commissions, or piece‐rate pay. However, for nearly half a century now, scholars have disagreed on how merit pay and other P4P practices affect employee motivation or effort and whether they enhance or undermine work performance (Chen et al., 2023). Indeed, some believe P4P undercuts employees’ intrinsic motivation for their jobs and can result in performance degradation, depending on the type of tasks, among other factors (Cerasoli et al., 2016; Weibel et al., 2010). We believe that one source of confusion in this debate is that very different forms of P4P are all lumped together. For example, a one‐time bonus because an employee handled a project exceptionally well, tells a different message and has a different financial impact than, say, yearly performance‐based permanent salary increases for all employees following an overall performance review, aka merit pay. Given that P4P practices with different characteristics are likely to have different effects (Nyberg et al., 2016), we believe they should be assessed separately. Therefore, with this systematic review, we want to evaluate the efficacy in enhancing employee work motivation, effort, and performance, of merit pay specifically, as it was the most commonly applied P4P practice over the past decades (Gerhart, 2023; Heneman, 1992; Rynes et al., 2005).

Rather than comparing merit pay to other P4P practices, we want to develop a more in‐depth understanding of the conditions and circumstances under which merit pay may be effective by taking into account intervention and study characteristics. More specifically, we will investigate whether merit pay predicts employee work motivation, effort, and performance by considering (a) all studies that assess the change in our outcomes of interest after a merit pay program was implemented or a merit increase was awarded, (b) studies which compare post‐intervention outcome levels with employees who are not subjected to a merit pay plan, or (c) studies which observe the association between the size of the merit increase and subsequent outcome levels while controlling for pre‐allocation levels of relevant variables. In addition to study type, we wish to investigate whether the association between merit pay and subsequent employee motivation, effort, and performance depends on the actual and the perceived relationship between performance and merit pay, as well as by the type of work motivation that was measured (i.e., general, intrinsic, or extrinsic).

### 1.2 The intervention

Merit pay is one of many types of compensation employers provide to their employees for their services. It is defined as an employee compensation system in which incremental increases in base salary are provided and where the provision or the size of the increase depends on a rating by the employer of the employee's past performance (Gerhart, 2023; Heneman, 1992). So merit pay usually involves a permanent increase in salary, which may be a fixed amount or more likely a percentage of the salary. Some organizations, on the other hand, award a lump‐sum bonus. In this case, the merit increase involves a one‐time raise that is not then continued as part of the base salary, often referred to as a merit bonus (Gerhart, 2023). The earlier definition, however, does not include awarding merit bonuses. For this review, we choose to only focus on merit pay as permanent salary increases, excluding merit bonuses, because they are different in nature and scope. With the latter, after all, the added pay needs to be re‐earned every year. In this respect, the motivational message of a merit bonus is quite different from that of merit pay (Gerhart, 2023). Moreover, a merit bonus, for example, 5% of one's annual salary, provides instantly a large sum that the employee can spend at will. In contrast, the same merit increase will be spread in smaller amounts over all paychecks for the following year. Meanwhile, a merit increase is worth much more given that the same increase is paid every year, as it is now part of the base salary (Nyberg et al., 2016).

The specific characteristics of merit pay also distinguish it from other forms of P4P. First of all, merit pay is normally based on rather subjective performance evaluations and not on objective or countable indicators such as sales or profit figures, as is the case with sales commissions or profit sharing. Second, individual performance is the basis for merit pay, not the performance of a group to which the employee belongs (e.g., team or business unit performance) (Heneman, 1992; Rynes et al., 2005). Third, it focuses on performance over some time, usually a year, and not on a performance that happened at a specific point in time. In the latter case, employers may award a lump‐sum bonus (Heneman, 1992; Nyberg et al., 2016). Finally, merit pay rewards actual performance, not potential performance. An example of rewarding potential is skill‐based pay, where employees are rewarded for acquiring skills that can help them perform better on the job (Heneman, 1992).

Furthermore, how organizations design and implement merit pay in practice, can also differ in several aspects. In the following paragraphs, we summarize the main variants based on the book on merit pay by Robert Heneman (Heneman & Werner, 2004; Heneman, 1992), which can be considered a reference work on the topic of merit pay. The first important aspect on which merit pay systems can vary is how employee performance is being measured. Typically, the direct supervisor is the only appraiser, but it certainly occurs that other parties also evaluate the employee, such as co‐workers, subordinates, or customers. In addition to the appraiser's identity, what is being appraised may also vary depending on the system. The performance standards against which the employee is rated may involve traits, behavior, competencies, goal attainment, results, or a combination. Here, the assessment of the employee's performance is usually expressed on a rating scale, but may also involve some countable indicators. To still speak of merit pay, in the latter case, it should not be about objective results, such as sales figures, but about criteria where the counting still includes a subjective judgment, for example, the number of successfully solved cases. Other than these absolute comparisons of the employee to a desired standard, some organizations use relative comparisons by ranking the employees within a group or through pairwise comparisons. Finally, while most organizations evaluate their employees once a year, some organizations do so with a different frequency, for example, bi‐annually, semi‐annually, or quarterly.

In addition to how they measure performance, merit pay systems may differ in the extent to which other factors than the performance appraisal determine the size of the salary increase. Indeed, the actual size of a salary increase for a specific performance level may depend on several factors such as the organization's available budgets and budgeting practices, job level, market adjustments, the width and the maximum of the pay range attributed to the job, or the position of the employee within that pay range (cf. the compa‐ratio). Usually, all employees within an organization receive their raises at the same time. However, some organizations use the anniversary of the employee's employment contract as the basis.

Lastly, merit pay systems may differ in how they are implemented and administered. Factors that seem to matter here, are the degree to which the performance criteria are tied to the strategic mission of the organization, upper management endorses the system, the system applies to all employees and not just a subgroup (e.g., managers), all rewards are made contingent upon performance and not just merit pay, employees are involved in the development of the merit pay plan, employees may appeal the performance evaluation awarded, sufficient and transparent information on the merit pay system is provided, supervisors are trained in making accurate and fair performance assessments, and legal restrictions must be respected. These factors also exemplify that merit pay should not be considered in isolation, but as part of a broader performance management system (Schleicher et al., 2018) and compensation policies (Nyberg et al., 2016).

### 1.3 How the intervention might work

While under a merit pay system salary increases are based on past performance, what it intends is to motivate increased performance in the future. The theories that have served as the underlying rationale for merit pay come from psychology and economics. Whereas theories from the two fields differ in their views on why, they all share the belief that linking pay to performance should increase employee work motivation or effort and, as a consequence, employee performance (Heneman & Werner, 2004; Heneman, 1992; Nyberg et al., 2016). Indeed, a key feature of merit pay is that it ties salary increases to employee performance. From a psychological perspective, this feature increases the instrumentality of high performance, which is the belief that high performance will be rewarded. Then, under the precondition that the expected salary increase is appreciated by the employee and that they expect that higher efforts will lead to better ratings, the belief that performance will be rewarded will motivate the employee to invest more effort in work. This line of reasoning follows from expectancy theory (Vroom, 1964), the most frequently used psychological theory to explain merit pay and other P4P practices (Nyberg et al., 2016), which states that an employee's motivation to perform depends on the belief that high performance will be rewarded (i.e., instrumentality), the perceived value of the reward (i.e., valence), and the expectation that increased efforts will lead to the desired performance (i.e., expectancy). Related to instrumentality, also reinforcement theory supports that linking salary increases to performance should increase employee performance (Heneman, 1992). After all, according to this theory, our behavior, including our performance, is determined by its consequences (Skinner, 1953).

Also from an economic perspective, it is believed that linking pay to desired performance should lead to performance benefits for the organization (Heneman, 1992; Nyberg et al., 2016). For instance, the incentive intensity principle (Milgrom & Roberts, 1992) suggests that to be effective an incentive should have an adequate level of intensity. One way to achieve this is to ensure that increased efforts lead to corresponding profits. Agency theory also suggests that when performance can be measured and pay is tied to performance levels, improved employee performance can be expected (Eisenhardt, 1989; Gerhart, 2023).

While the previous theories state that employee motivation depends on the association the employee perceives between pay and performance, equity theory states that it will also depend on comparing this association with their peers. According to equity theory (Adams, 1965), employees believe that pay should be given based on how much each contributes. It suggests that merit pay will increase employee motivation if the ratio of inputs (e.g., effort, performance) to outputs received (e.g., merit increases) is perceived as equitable, which will result in higher performance. Employees will experience this equity when the ratio of their perceived outputs to inputs equals the perceived output to input ratio of peers with whom they compare themselves. When they believe to be under‐rewarded or over‐rewarded, however, they will be driven to restore this imbalance. The ensuing actions will often not benefit the organization, for instance, lower efforts, neglect of quality, lawsuits, or turnover. Therefore the goal of a merit pay system is to make sure the association between salary increases and performance is perceived as equitable (i.e., “who contributes more, gets paid more, so everyone is treated fairly”) so that employees are not encouraged to engage in behavior that may be detrimental to the organization (Chen et al., 2023; Gerhart, 2023; Heneman, 1992).

Furthermore, next to incentive effects, merit pay may also have so‐called sorting effects (Gerhart, 2023). In other words, paying employees based on their so‐called marginal productivity or performance is expected not only to incentivize increased efforts but also to attract and retain employees who want to be paid according to their performance and work hard to maximize their outcomes (Bishop, 1987; Heneman, 1992). People who do not like such a system or perform weakly will not accept or leave a job in such organizations. So it is believed that merit pay affects employee performance not only directly, but also indirectly through employee attraction and attrition.

In addition to the theories described above, other theories have been used as well to explain how merit pay works. However, these seem to be less frequently cited in relation to merit pay and therefore we do not explain them further here. These include goal‐setting theory, implicit contract theory, efficiency wage theory (see Heneman, 1992), and tournament theory (see Park & Sturman, 2016). Finally, some theories question whether merit pay motivates at all and will lead to better performance (Campbell et al., 1998; Heneman, 1992). We address this point of debate in the following section on why it is important to do this review when discussing the rationale for our research questions.

### 1.4 Why it is important to do this review

#### 1.4.1 Current syntheses on merit pay

Merit pay has been referred to as the grandfather of all P4P plans and as one of the most commonly applied employee compensation systems over the past decades (Gerhart, 2023; Heneman, 1992; Rynes et al., 2005). To the best of our knowledge, however, there are no systematic reviews on the overall effectiveness of merit pay at present.

Two reviews, one meta‐analysis (Pham et al., 2021) and one nonsystematic literature review (Kim, 2021), although based on their title they seem to pretend to evaluate the use of merit pay within education, actually focused on different P4P practices in general. Indeed, merit pay is sometimes inappropriately used as a synonym for P4P. Pham et al. (2021) explicitly stated that merit pay programs are “also called performance pay, pay for performance, or differentiated pay” (p. 528), which already reveals that their focus was broader than merit pay in its strict sense. Also from the program characteristics that were coded by the authors, we can infer that not all included studies involved merit pay, namely whether the program provided incentives to groups of teachers and whether the criteria for meeting the bonus eligibility threshold were based on test scores and at least one other performance measure (e.g., observation scores or student perception surveys). Student test scores is an objective performance indicator, which does not rhyme with merit pay. Of the 41 studies included in the meta‐analysis, 13 studies involved a group incentive structure. In table 2 of their paper, the authors state that they identified different award types in the studies, namely not only salary increases, but also bonuses or gifts. Further, the paper does not provide sufficient detail to determine in how many studies there was a true merit pay program, let alone report an effect size and other statistics for this group of studies. Kim (2021), as well, states that merit pay is “also known as performance‐based pay” (p. 2). Of the 13 studies that he reviews in his paper, at least 5 of them seem to involve bonuses, not permanent salary increases, and/or objective performance indicators like student test scores (Clotfelter et al., 2008; Coates‐McBride & Kritsonis, 2008; Figlio & Kenny, 2007; Ritter et al., 2008; Sojourner et al., 2014, all as cited in Kim, 2021). On other studies, Kim (2021) shares too little information to determine whether it is really about merit pay. Thus, these reviews do not provide accurate conclusions about merit pay specifically.

Other existing meta‐analytic reviews around P4P or incentives in general mention merit pay as an example of P4P, but do not detail how many studies specifically involved merit pay as an intervention, nor do they report results specific to this type of P4P (Chen et al., 2023; Weibel et al., 2010), or (and in most cases) the term merit pay does not even appear in the text (Cerasoli et al., 2014; Cerasoli et al., 2016; Condly et al., 2003; Garbers & Konradt, 2014; Jenkins et al., 1998; Kim et al., 2022). In sum, over the past few decades these reviews presented contradictory findings on the extent to which P4P or incentives always reinforce performance or to varying degrees according the performance indicator (e.g., quantity, quality, task performance, contextual performance), the type of task (e.g., non‐interesting vs. interesting, low vs. high complexity, ability vs. motivation‐driven), and other moderators (e.g., study design or setting, incentive intensity). In addition, debates also rage on the extent and circumstances under which P4P and other incentives frustrate intrinsic motivation and its relationship to performance and whether this differs depending on the characteristics of the incentive (e.g., contingency, saliency) or other factors (e.g., performance type). Merit pay is indeed an incentive and a form of PFP, but none of the existing meta‐analytic reviews report separate effect sizes specifically for merit pay. Moreover, most of these meta‐analyses consist mainly of lab experiments or experimental simulations, or include mostly nonwork contexts (e.g., school, physcial domains). The question, then, is to what extent these findings can be generalized to real‐world merit pay programs.

In his textbook on employee compensation, Gerhart (2023, p. 325) refers to Heneman (1992) reporting that 40 of 42 studies looking at merit pay show higher performance when pay is tied to performance, as evidence for the efficacy of merit pay as a P4P practice. However, Heneman (1992, p. 47) explicitly points out that the positive relationship in these studies involves that between merit increases and previous performance levels. This only shows that organizations can be successful at tying pay to previous performance because they do indeed succeed in establishing a correlation between salary increases and the performance ratings on which they are based. The question is whether this link will lead to favorable outcomes. Stated differently, to demonstrate whether merit pay is effective, one should be able to show that the allocation of a merit increase or the size of the merit increase is associated with subsequent performance while controlling for prior performance levels. Without controlling for previous performance levels, any merit pay effects could be interpreted as merely indicating that better performers tend to both receive (higher) merit increases and perform well in the future.

In their nonsystematic review of performance evaluation and various P4P practices, Rynes et al. (2005) concluded over 18 years ago that there is surprisingly little evidence of merit pay's efficacy in terms of improving employee performance and called for more well‐designed studies on its efficacy. They mention that Heneman (1992, pp. 247–252) lists in his book on merit pay 10 studies of limited internal validity that investigated the performance impact of merit pay and reports that the results were positive and significant in half of the studies. In the other half, the effect sizes were not significant. No statistically significant negative associations were reported. The fact that merit pay had not consistently been related to employee motivation or performance and the limited internal validity of the existing studies, led Heneman (1992) to conclude that the assertion that merit pay plans are effective could at best be tentative. He also added that we need more research that can uncover the factors that determine when merit pay has favorable outcomes. With this review, we want to collect all new studies that have appeared since the call from Rynes et al. (2005) for more research on merit pay and critically synthesize what they, together with prior research, can teach us about the effectiveness of merit pay today.

#### 1.4.2 Relevance for practice and policy

Understanding whether and when merit pay is effective has important implications for practice and policy. Organizations implement motivation‐enhancing HR practices, which often include performance‐based employee compensation systems such as merit pay, to promote employee motivation and, in turn, firm performance (Jiang et al., 2012). By their nature, merit pay systems may over time be associated with significant increases in fixed‐wage costs (Gerhart, 2023). Given the wide adoption of this pay practice and the undeniable costs that go with it, a review of its efficacy should be of great importance to organizational decision‐makers. Governments as well, under budgetary pressures and public opinion demanding a more modern and efficient civil service, have increasingly considered the implementation of some form of performance‐related pay. In fact, during the past decades, an increasing number of OECD countries have introduced and maintained P4P in the government ranks to some extent (OECD, 2005, 2017). At the same time, the effectiveness of P4P within the public sector has been debated (Hasnain et al., 2014; Jordan & Battaglio, 2014; Park, 2022; Weibel et al., 2010), which again warrants a systematic review related to the issue.

#### 1.4.3 The rationale for our research questions

Our primary research question is whether merit pay is positively associated with subsequent employee work motivation, effort, and performance. Given the mixed findings in previous research (Heneman, 1992, pp. 247–252), we expect on average no more than weak positive associations between merit pay and subsequent motivation, effort, and performance, as well as a certain degree of heterogeneity in the effect sizes. We predict this heterogeneity may in part be explained by three different moderators, translated into three additional research questions (see Objectives). All three have practical relevance as they can provide more insights into the circumstances under which merit pay may result in positive outcomes and how merit pay may affect employees.

The first and second moderators relate to the assumption that the efficacy of a merit pay system depends on the degree to which it manages in practice to establish a clear “line‐of‐sight” between work performance and pay (Nyberg et al., 2016; Schaubroeck et al., 2008). Indeed, the absence of a clear relationship between performance and salary increases is a violation of the underlying rationale for merit pay. In this respect, both the actual and perceived relationship between performance ratings and merit increases are believed to be pertinent (Heneman & Werner, 2004; Heneman, 1992; Nyberg et al., 2016; Schaubroeck et al., 2008). Therefore, the first moderator we wish to consider in this review is the actual relationship between the employee performance ratings and the subsequent merit pay increases. After all, it is less likely that employees will believe that higher performance leads to higher merit pay increases if, in reality, both variables do not correlate or do so only weakly. And, it has indeed been observed that this correlation is weak in a substantial number of cases (Heneman, 1990, as cited in Heneman, 1992). This means that in some organizations other factors primarily determine the size of merit pay increases, for example, available budgets, seniority, or cost of living adjustments (Heneman, 1992). The second moderator we wish to consider is the perceived relationship between performance and merit pay. In other words, the effectiveness of merit pay is expected to depend on how strong employees perceive the association between pay and performance to be (Nyberg et al., 2016; Park & Sturman, 2016; St‐Onge, 2000). Indeed, various factors are thought to affect the P4P perceptions, such as the measurability of performance (Milgrom & Roberts, 1992; Nyberg et al., 2016), the transparency of the P4P system (Wenzel et al., 2019), trust in the decision‐makers involved, or the perceived procedural justice of the decision‐making process (St‐Onge, 2000).

With the third moderator, we limit our focus to the association between merit pay and work motivation. Work motivation can be defined as: “a set of energetic forces that originate both within as well as beyond an individual's being, to initiate work‐related behavior, and to determine its form, direction, intensity, and duration” (Pinder, 2008, p. 11). One implication of this definition is that motivation can originate from different sources (Pinder, 2008). An important distinction that is made at this level in the context of P4P (Gerhart & Fang, 2014; Hasnain et al., 2014; Rynes et al., 2005; Weibel et al., 2010), is the one between intrinsic motivation, which refers to “doing an activity for the inherent satisfaction of the activity itself” (Ryan & Deci, 2000, p. 71), and extrinsic motivation, which refers to “the performance of an activity to attain some separable outcome” (Ryan & Deci, 2000, p. 71). Now, some challenge the presumed incentive effects of merit pay, as merit pay, being an extrinsic motivator, may reduce intrinsic motivation and, consequently, the nature of employee efforts and performance (Campbell et al., 1998; Heneman, 1992). Indeed, proponents of this view refer to the cognitive evaluation theory (Deci & Ryan, 1985) stating that merit pay may undermine intrinsic motivation because of its controlling nature, forcing employees to meet some performance standards before they can earn a pay raise. However, tapping on the same theory, recent research suggests that P4P in organizations, and thus merit pay as well, may also play an informing role in which receiving merit increases tells employees that they are doing a good job, which increases their feelings of being competent and their intrinsic motivation for their work (Chen et al., 2023). For this reason, the third moderator we consider in this review is the type of motivation that was measured (i.e., general work motivation, intrinsic motivation, or extrinsic motivation), which will also allow us to clarify to which degree merit pay seems to undermine or strengthen intrinsic motivation.

We expect that the association between merit pay and subsequent employee motivation will not depend on motivation type. In other words, we do not expect this association to be weaker or negative for intrinsic motivation. In their meta‐analysis, Chen et al. (2023) found that P4P enhanced intrinsic motivation. Also Cerasoli et al. (2014) reported in their meta‐analysis that incentives were associated with higher levels of intrinsic motivation, but only if the incentives were indirectly salient to performance. Incentives directly salient to performance were associated with lower levels of intrinsic motivation. For directly salient incentives such as piece‐rate pay, sales commissions, or end‐of‐year bonuses, there is a clear and narrow link between different performance levels and the size of the incentive received. This is also the case with merity pay, but here performance is determined through a subjective evaluation and not by countable indicators such as pieces, sales or profit figures. Moreover, those performance reviews consider usually a range of different indicators that require subjective judgments (e.g., competency ratings) and also aims to judge overall performance throughout the year instead of results at a specific point in time. In other words, merit pay is much less controlling than directly salient P4P practices. It does not spur and confine employees to aiming for specific results, but we expect it to motivate them to be good at their job in all its aspects and with a certain amount of freedom. Another meta‐analysis by Cerasoli et al. (2016) provided evidence that indirectly salient incentives might indeed boost employees’ need to feel autonomous and competent in their work.

Finally, besides the previously discussed moderators, we expect the observed association between merit pay and our outcomes of interest might also be (inadvertently) attenuated in some of the studies by sorting and ceiling effects. As explained earlier, sorting effects can indeed be expected with merit pay (Park & Sturman, 2016; Rynes et al., 2005). As a merit pay system attracts (motivated) high performers and divests (demotivated) low performers, mostly motivated, high performers will remain in the organization. This reduction in the variance of motivation, effort, and performance may weaken the observed association between merit pay and subsequent performance, especially in organizations where the system has been in place for some time. For the so‐called ceiling effects, we draw attention to the nature of the scales used in studies to rate our outcomes of interest. In merit pay systems performance is often appraised by using rating scales. Hereby appraisers can indicate for each performance standard what label, usually on a 5‐point scale, for example, from well below average to well above average, that best describes the employee's performance level. Overall performance is then either calculated by taking the (weighted) average or it is discretionarily, and thus more subjectively, determined by the rater (Gerhart, 2023). As a consequence, especially in the latter case, employees who receive the highest performance rating, cannot receive a higher rating in subsequent years. Meanwhile, it is reasonable to expect that at least for some their performance nonetheless still improved compared to previous years (Sama et al., 1994). Moreover, recent research has shown that worker productivity in 21st‐century knowledge‐based organizations may be following a distribution in which the highest performance differences are observed among the star performers within an organization and not around the average (Aguinis & O'Boyle, 2014). These differences are not well captured then by traditional rating practices in which all the star performers receive the highest rating. So all things considered, when controlling for previous performance levels, the variability in subsequent performance levels will be reduced, likely weakening its association with merit pay, a so‐called ceiling effect (Wang et al., 2008). In the same vein, ceiling effects may also be expected for employee motivation and effort, as they are traditionally measured with Likert‐type scales with a limited number of answer options (Hessling et al., 2004).

As the potential sorting effects in merit pay studies pertain to the selection of participants into the studies and the potential ceiling effects to the measurement of outcomes, they should both be considered as potential risks of bias that may be addressed differently from study to study (Sterne et al., 2016). Therefore, we do not formulate separate research questions for this review around these, but they will of course be assessed at the level of each included study, and if appropriate, we will test whether there is an association between our risk of bias assessments and the effect sizes. In other words, a final contribution of this review is to clarify to which degree conclusions about merit pay might be biased because of different methodological aspects.

## 2 OBJECTIVES

2.1

The research questions that this review aims to answer are

1. Does merit pay predict employee work motivation, effort, and performance?
2. Is the association between merit pay and subsequent employee work motivation, effort, and performance stronger depending on the actual relationship between the performance ratings and merit increases received?
3. Is the association between merit pay and subsequent employee work motivation, effort, and performance stronger depending on the perceived relationship by employees between their performance and their pay?
4. Does the association between merit pay and subsequent employee motivation depend on what type of motivation is measured (i.e., intrinsic vs. extrinsic/general work motivation)?

## 3 METHODS

### 3.1 Criteria for considering studies for this review

#### 3.1.1 Types of studies

Our primary research question is a prediction question because we do not expect to identify research designs that will allow us to make assertions about the effects of merit pay. Indeed, we do not expect to find randomized controlled trials, since we focus on organizations and how their compensation practices affect their employees. Field experiments are rare in organizational psychology and organizational behavior research (Eden, 2017). In most cases, it would be considered unethical or unfeasible to randomly assign employees to different treatment conditions and deliberately manipulate their paychecks, granting higher salary increases to some employees while withholding them from others (Grant & Wall, 2009; Lawler, 1977). So, we expect to find only non‐randomized studies on the effects of merit pay.

Adequate study designs for addressing our prediction question are all quantitative study designs that can establish an association between our variables of interest and where the measurement of our dependent variables (i.e., employee work motivation, effort, and performance) happened after the application of merit pay, including field experiments (i.e., randomized studies), quasi‐experiments (e.g., natural experiments), before‐after studies, and longitudinal studies (e.g., repeated measures studies), and case‐control studies. Moreover, eligible studies will use controls for (a) levels in our dependent variables as observed before a merit increase was awarded, (b) the employee's actual pay levels, and/or (c) include comparison groups in which a different compensation system is applied. Finally, cross‐sectional and qualitative study designs will be excluded.

##### Types of participants

The subjects in eligible studies are part‐time or full‐time employees or workers of private or public organizations. We do not exclude any worker populations based on region, sector, organization level, permanent versus temporary employment, ethnicity, or other demographic characteristics. However, studies conducted with nonworker populations like volunteers, students, job seekers, incapacitated persons, retirees, or self‐employed (e.g., contractors or freelancers) are excluded. We also exclude studies conducted with workers who perform their work at an organization but are employed by a third party, such as agency workers or leased workers. After all, the nature of the work situation of these workers, such as the level of feelings of job insecurity, perceived employability, or pay conditions, is very different from traditional workers who have a direct working relationship with the organization where they perform their work, which may affect our outcomes of interest (e.g., through reduced satisfaction) (Spreitzer et al., 2017).

##### Types of interventions

The intervention in eligible studies should fit the definition of merit pay as an employee compensation system in which incremental increases in base salary are provided and where the provision or the size of the increase depends on a rating by the employer of the employee's past performance (Gerhart, 2023; Heneman, 1992). This definition excludes studies on merit bonuses, in which the reward involves a one‐time raise, aka a lump‐sum bonus, that is not then continued as part of the base salary. Studies that qualify either report for each participant whether or not they are subject to a merit pay program, whether or not they have received a merit increase, and/or the merit increase size. Other P4P systems such as merit bonuses, incentive pay, or collective P4P plans are not the focus of this review unless they are provided in a comparison condition. In addition to alternative forms of P4P, any employee compensation system is considered a relevant comparison condition, for example, a system whereby incremental salary increases are based on seniority.

##### Types of outcome measures

###### Primary outcomes

The primary outcomes in this review are individual‐level (i.e., employee) measures of work motivation, work effort, and work performance. These outcomes are conceptually and empirically closely related, but distinct from each other (Van Iddekinge et al., 2023). Some scholars, however, have treated work effort and motivation as identical, but Van Iddekinge et al. (2023) argued and empirically demonstrated that work effort should be considered as a direct outcome of motivation. They define work effort as “How hard workers try to perform their jobs, which includes where they devote their effort (direction), the amount of their effort (intensity), and how long they persevere in their effort (persistence)” (p. 6). With this definition, they consider work effort also as a direct antecedent of employee performance. Indeed, they stated and evidenced that “effort is necessary, but insufficient, for performance in that workers need to exert effort to perform their roles, but their effort must be efficient and focus on the appropriate behaviors” (Van Iddekinge et al., 2023, p. 20).

Although work effort has been defined in terms of direction, intensity, and persistence, in most studies the measures of effort focus on intensity and/or persistence, or they asses it in a general way. Measures of the direction of effort are considered rare (Van Iddekinge et al., 2023). We consider all measures of effort eligible for this review, whether they focus on effort in general, a specific dimension, or multiple dimensions. Meanwhile, in the merit pay literature, we could already identify several studies using the behavioral intention measure of Mitra et al. (1997) which asks respondents, “What effect will the change in your pay between last year and this year have?” In response, they can express on a 7‐point scale how hard they will work compared to before (e.g., “I will work a lot harder than before.”). At first glance, this measure could be considered a general measure of work effort. However, since it measures one's intentions to work harder and not actual effort expended, it should be considered a general measure of work motivation. Indeed, according to the theory of planned behavior, “intentions are assumed to capture the motivational factors that influence a behavior; they are indications of how hard people are willing to try, of how much of an effort they are planning to exert, to perform the behavior” (Ajzen, 1991, p. 181). Other measures of motivation we consider eligible for this review are alternative measures of general work motivation and specific measures of intrinsic or extrinsic motivation, which we have defined in the background section of this review.

Finally, work performance is defined as “things that people actually do, actions they take, that contribute to the organization's goals” (Campbell & Wiernik, 2015, p. 48). In organizational research, employee performance is most commonly measured through performance ratings. The rating source can be the employee themself, or others, such as supervisors, peers, or subordinates. Alternatively, employee work performance can in some cases also be measured through more objective indicators or outcomes, such as sales figures, production rates, or goal achievement (Campbell & Wiernik, 2015; Sonnentag et al., 2008). We consider both subjective and objective measures of performance eligible for this review. We also do not exclude measures of performance based on their content, whether they focus on general work performance, core task performance, or contextual performance and related constructs (e.g., organizational citizenship behaviors, change‐oriented behaviors). The latter refers to behaviors that often go beyond what is formally required by the job description, being more discretionary, proactive, or change‐oriented, and do not necessarily directly contribute to organizational performance but support the effective functioning of the organization, including on a social and psychological level, for example, volunteering, helping others, taking initiative or compliance (Campbell & Wiernik, 2015; Marinova et al., 2015; Sonnentag et al., 2008).

###### Secondary outcomes

While our primary outcomes for this systematic review focus on the individual level, the secondary outcomes, if available, will be work motivation, effort, and performance measured at a group level, which can include the team level, unit level, or organization level.

###### Duration of follow‐up

We will apply no restrictions based on the duration of follow‐up.

###### Types of settings

The study must have been conducted in an organizational, real‐world setting and not within a lab or simulated context.

### 3.2 Search methods for identification of studies

To address our questions about the association between merit pay and employee work motivation, effort, and performance, we developed a search strategy that will help to delineate our search of the worker compensation literature. Our search strategy aims to limit the search results to the following output:

- Studies focused on merit pay.
- Studies in which employee work motivation, effort, or performance are measured. The search strategy will be used in several multi‐disciplinary, subject‐specific, and regional or language‐specific bibliographic databases, as well as gray literature repositories and search engines. In the bibliographic databases, we will use keywords to find relevant studies combined with subject headings when available. These searches will be limited to titles, abstracts, keywords, and controlled vocabulary. The gray literature repositories will be consulted through keyword searching, when possible. The subject headings and keywords will relate to merit pay, being the intervention of interest, as well as work motivation, effort, and performance, the outcomes of interest. For some databases or repositories, we will limit the search query to intervention‐related terms when this results in a feasible number of titles and abstracts being screened, which will increase the sensitivity (i.e., comprehensiveness) of our search strategy. Relevant studies will also be collected by reviewing for inclusion the reference lists and citations, as per Google Scholar, of included studies and related literature reviews. We will also contact the first authors of the included studies to ascertain whether they are aware of any additional published, unpublished, or ongoing studies.

#### 3.2.1 Electronic searches

We will consult a selection of multidisciplinary, subject‐specific, and regional or language‐specific bibliographic databases. The multidisciplinary bibliographic databases we will consult are:

- Directory of Open Access Journals.
- Scopus (via Elsevier).
- Web of Science Core Collection (via Web of Science).

The subject‐specific bibliographic databases are:

- ABI/Inform Collection (via Proquest).
- APA PsycINFO (via EBSCOhost).
- Business Source Complete (EBSCOhost).
- Research Papers in Economics (RePEc) (via via EconPapers).
- Social Science Premium Collection (via Proquest).
- Social Science Research Network.

The regional or language‐specific bibliographic databases are:

- Cairn.
- China Journals, China Doctoral Dissertations & China Master's Theses, and China Proceedings of Conference (via CNKI).
- KCI‐Korean Journal Database (via Web of Science).
- SciELO Citation Index (via Web of Science).

We will apply an adapted search strategy for each of these databases, depending on the available search fields, operators, subject headings, and the applicable language. When available, limiting commands will be applied to exclude irrelevant document types, such as wire feeds, trade journals, newspapers, industry profiles, country reports, product reviews, etc. We will apply no limiting commands related to the publication date or the language of the study, as all are considered relevant. The search strategy specific to each database can be retrieved in the supplementary materials (see Appendix S1). We may also search additional databases during the search process should that prove appropriate.

#### 3.2.2 Searching other resources

##### Gray literature

Where possible, we will also include gray literature when consulting the bibliographic databases already listed under the previous section. Thus, the databases, ABI/Inform Complete, APPA PsycINFO, Business Source Complete, Web of Science, China Doctoral Dissertations & China Master's Theses, China Proceedings of Conference, RePEc, and Social Science Premium Collection, should also provide dissertations, theses, conference papers, proceeding papers, meeting abstracts, working papers, market research reports or electronic collections. To identify additional relevant gray literature, we will also consult the following sources:

- Bielefeld Academic Search Engine (BASE): https://www.base-search.net/Search/Advanced—We will filter for Document Types: Conference object, Report, and Thesis.
- Digitala Vetenskapliga Arkivet (DiVA): https://www.diva-portal.org/‐ We will filter for Student theses.
- Global ETD (electronic theses and dissertations) Search—http://search.ndltd.org/.
- Google: https://www.google.com/—The following keywords will be added to the search string: conference, proceeding, meeting, dissertation, thesis, “working paper.” Searches will be limited to the top 100 hits, sorted by relevance.
- Harvard Business Review: https://hbr.org/.
- National Bureau of Economic Research Working Papers: https://www.nber.org/papers.html.
- National Center on Performance Incentives: https://my.vanderbilt.edu/performanceincentives/.
- Open Access Theses and Dissertations (OATD): https://oatd.org/.
- Proquest Conference Papers & Proceedings (Proquest): https://www.proquest.com/.
- ProQuest Dissertations & Theses Global (PQDT Global).
- Proquest Working Papers (Proquest): https://www.proquest.com/.
- Society for Human Resource Management (SHRM): https://www.shrm.org/.
- U.S. Bureau of Labor Statistics: https://www.bls.gov/.
- WorldatWork Total Rewards Association (formerly American Compensation Association): https://worldatwork.org/.
- E‐reward.co.uk: https://www.e-reward.co.uk/.

The specific search strategy for each database or repository will be detailed in the appendices of the review. We may also search additional databases, websites, or repositories during the search process should that prove appropriate.

##### Citation searches and contacting authors

To identify additional, potentially relevant studies, we will also screen the reference lists of retained studies, as well as their citations. We will also screen the reference lists of the previously mentioned reviews on teacher merit pay (Kim, 2021; Pham et al., 2021) for eligible studies, as well as the reference list of Heneman's books (Heneman & Werner, 2004; Heneman, 1992) on merit pay. Finally, we will also contact the first authors of the included studies to ascertain whether they are aware of any additional published, unpublished, ongoing, or in‐press studies. In addition, we will also launch a call for unpublished work by posting on the discussion boards of relevant communities of the Academy of Management (e.g., Human Resources Devision, Organizational Behavior) and possibly other listservs as well.

##### Hand searches

For the journals that delivered at least two or more eligible studies, we will search the table of contents of the past year's issues given these may not yet all be indexed in one of the bibliographic databases.

### 3.3 Data collection and analysis

#### 3.3.1 Description of methods used in primary research

As labels, such as “cohort study” or “longitudinal study,” are inconsistently applied and can refer to many different study designs, we follow the recommendation in the Cochrane Handbook for Systematic Reviews of Interventions to avoid using these labels (Sterne et al., 2022). For assessing the risk of bias in non‐randomized studies, however, the handbook does make a distinction between three broad categories, namely uncontrolled before‐after studies, controlled before‐after studies, and follow‐up studies, because they require taking into account different aspects. We will discuss the common design features of studies on merit pay in line with this distinction.

The first category of study designs we expect to find in eligible studies is uncontrolled before‐after studies. In these studies, all participating employees are subject to a merit pay system, and the outcomes of interest are measured at least once before the implementation of the merit pay system and at least once sometime after the implementation. One example is the study by Deckop and Cirka (2000) who tested whether there was a change in intrinsic motivation at Time 2, as compared to Time 1, after the implementation of a merit pay system. Other uncontrolled before‐after studies, which can be considered a different type of study design, do not evolve around the implementation of a merit pay system, but around the issuing of a merit increase at a specific point in time (e.g., Park & Sturman, 2016). In these cases, it may be assumed that merit increases were already awarded periodically in the organization before the study was taken. In these studies, the authors do not test whether there was a change in the outcome of interest after a merit increase was awarded, but whether the size of the increase is associated with the outcome at Time 2 (e.g., future performance) while controlling for the outcome and optionally other potential confounds measured at Time 1 (e.g., past performance). In a third type of study design within this category, the authors test the same association, but across multiple time points and successive merit increases. For instance, over a 5‐year period, Nyberg and his colleagues (Nyberg et al., 2016) tested whether there was a positive association between the merit increase received in 1 year and employee performance in the following year while controlling for the average performance in the previous years and other confounds.

Second, we also expect to identify so‐called follow‐up studies (Sterne et al., 2022) in which participants are followed up (retrospectively) from the start of the merit pay program or the awarding of a merit increase up to a later time for measurement of the outcomes of interest. In such studies, the employees who are subjected to a merit pay plan are compared to employees who are not. Dee and Keys (Dee & Keys, 2004), for instance, compared the maths scores and reading scores of students who were randomly assigned to either a teacher who voluntarily enrolled in a merit‐pay plan or one who decided not to do so. In another type of study, the researchers test again whether the size of a merit increase is associated with an outcome measured after it was awarded, but only control for baseline confounds and not for pre‐award levels of the outcome. Shaw and his colleagues (Shaw et al., 2003), for instance, tested the association between the size of a merit increase and the employee's intention to work harder thanks to their change in pay while controlling for baseline measurements of pay‐level satisfaction, job level, age, gender, tenure, pay level, P4P perceptions, and positive affectivity.

Finally, although we have not identified such examples yet, we may also expect to find controlled before‐after studies. These would be studies in which (1) employees are non‐randomly allocated to a group that is subjected to a merit pay system or to an alternative group that is subjected to a different compensation system, and (2) at least one measurement of our outcomes of interest is made in both groups before and after the implementation of the merit pay system.

As mentioned earlier, the primary intention of merit increases is to motivate increased performance in the future. As it is our objective to test this assertion, it is also important to consider how employee work performance is measured in organizational psychology and organizational behavior research. This happens most commonly through performance ratings, the rating source being the employee themself, or others, most often the supervisor, or peers (Campbell & Wiernik, 2015; Sonnentag et al., 2008). It is considered a serious risk of bias when the outcome was assessed by assessors aware of the intervention received by study participants (Sterne et al., 2016), here being whether and what merit increase the employee received. However, we predict that this will often be the case in studies on merit pay. Therefore bias in the measurement of the outcomes is an inherent issue in this research domain. Indeed, meta‐analytic evidence shows that employees’ self‐ratings of performance are more lenient when they are collected for administrative purposes (i.e., compensation and promotion‐related decisions) compared to research purposes (Heidemeier & Moser, 2009). meta‐analytic evidence that led to the same conclusion was also collected for supervisor ratings of employee work performance (Jawahar & Williams, 1997). A key focus in assessing merit pay studies will, therefore, be to map whether the researchers collected performance ratings separately for the communicated purpose of research or whether they relied on company records of performance ratings that were likely collected for administrative purposes. Yet, even when research‐based performance ratings are being collected, we should keep in mind that the assessor will potentially rely on the memory of the merit increase awarded in the past to make a judgment of the current level of performance, since it is, after all, based on a previous performance rating. In other words, previous performance ratings of an employee related to the allocation of a merit increase may somehow bias the assessor's judgment of their current performance. One possible solution to this problem is, of course, the use of objective performance indicators, but we expect that the number of studies doing so will be small.

#### 3.3.2 Selection of studies

All the results from the electronic database searches and the searches of other resources will be added to Covidence, a software program for carrying out systematic reviews. For Covidence will be used for the deduplication and the screening of the results. The duplicates that will be removed by Covidence, will be checked by the first author to see if any references were erroneously removed by the software. Next, all results will be independently screened by two review authors through a process consisting of two phases, the initial screening and the full‐text screening. At the initial screening, irrelevant material will be excluded based on a review of the titles and abstracts. A screening key has been developed that guides the reviewers through several questions (see Appendix S2). A reference will be excluded once “no” constitutes the answer to one of the questions. As soon as one of the two review authors answers all questions about the reference affirmatively or answers one question with “unsure” (i.e., because the information in the title or abstract is insufficient or unclear), the reference will be withheld for the next phase. The screening key will be pilot‐tested by the two review authors based on the screening of a minimum of 120 references and will be adjusted as necessary.

The second phase involves screening each reference for eligibility based on a consultation of the full text, again independently by two review authors. Also for this phase, a screening key has been developed (see Appendix S2) and will be pilot‐tested based on the screening of the full text for a minimum of 10 references. When a reference is excluded based on the consultation of the full text, the reviewer will record the reason for exclusion. Disagreements between the two reviewers will be discussed and resolved by consensus. The authors of the paper will be contacted when information is missing to make an accurate decision.

#### 3.3.3 Data extraction and management

A data extraction and coding form have been developed in Covidence (see Appendix S3), the platform in which the data will also be stored. Data and information will be extracted on sample characteristics, study setting, contextual features, intervention characteristics, study design, theoretical foundation, outcome measures, results, and risks of bias. For coding data on the design of a study, we based our data extraction and coding form on the quasi‐experimental taxonomy features checklist, as to avoid the ambiguities of study design labels (Reeves et al., 2017). Two review authors will pilot‐test the coding form based on the extraction of three studies and adjust it as necessary in consultation with the other review authors. Next, all other review authors who will participate in the data extraction will be trained in the use of the coding form. Finally, two review authors will be assigned to each eligible study for independent data extraction. Disagreements between two reviewers will be discussed and resolved by consensus, and subject‐matter experts will be consulted when appropriate. These disagreements and their resolution will also be reported. Moreover, if an eligible study is in a language that none of the review authors understand, we will have the paper translated or look for researchers who are proficient in the language and can extract the data in consultation with a review author. Finally, the authors of the paper will be contacted when important information is missing.

#### 3.3.4 Assessment of risk of bias in included studies

As discussed in the background section of this protocol, we will pay particular attention to whether and how potential sorting and ceiling effects were addressed in each study. To assess these and other risks of bias in the included studies, we will use the ROBINS‐I tool (Risk Of Bias In Non‐randomized Studies ‐ of Interventions) (Sterne et al., 2016). This tool focuses on the internal validity of studies and considers seven domains of bias, namely (1) bias due to confounding, (2) bias in the selection of participants into the studies, (3) bias in the classification of interventions, (4) bias due to deviations of intended interventions, (5) bias due to missing data, (6) bias in the measurement of outcomes, and (7) bias in the selection of the reported results. Based on answers to a set of signaling questions, judgments for each bias domain, and overall risk of bias will be formed, per outcome of interest included in the study. Two review authors will independently assess the risk of bias for each included study. Disagreements between the two reviewers will be discussed and resolved by consensus, and a third review author or subject‐matter experts will be consulted when appropriate. These disagreements and their resolution will also be reported.

The ROBINS‐I tool (Sterne et al., 2016) recommends already listing at the protocol stage of systematic reviews potential important confounding domains relevant to all or most studies eligible for the review. Relevant confounding domains are the prognostic factors (i.e., factors that predict the outcome of interest) that predict whether an individual receives one or the other intervention of interest. In other words, these are factors that predict employee work motivation, effort, or performance, and predict whether and what merit increase an employee will receive. Since the allocation of merit increases is based on ratings of past performance, an important confound is the pre‐allocation levels of our outcomes of interest. Indeed, employee work performance is predicted by employee motivation, effort (Van Iddekinge et al., 2023), and past performance (Sturman et al., 2005).

A second confounding domain is related to the group of factors that influence the actual size of the merit increase that is allocated for a specific performance level. For instance, merit increases are often calculated according to a percentage of base salary. Moreover, in some cases, the applied percentage may even depend on their current position within a pay range defined for that job (Heneman & Werner, 2004; Heneman, 1992). So the absolute value of the raise depends on the employee's base salary level. Simultaneously, we may also expect that an employee's base pay or satisfaction with the pay level is associated with their work motivation, effort (Kuvaas et al., 2016), and performance (Williams et al., 2006). We should also take into consideration other factors that are associated with an employee's pay level and may therefore predict the size of their merit increases, namely the employee's seniority or organizational tenure, job level (e.g., managerial vs. nonmanagerial, exempt vs. nonexempt), education level, and the industry in which they work. Specifically in longitudinal designs with measures of our outcomes of interest across multiple time points and successive merit increases, another potential confounding factor is whether the employee got promoted during the study period, as this can affect their pay level and performance ratings (Nyberg et al., 2016).

In the same vein, a third confounding domain is related to the group of factors that influence whether the employee will receive a favorable performance rating and, therefore, merit increase. Factors that come into focus again here are organizational tenure, job level, and education level, as they have been associated with more favorable performance ratings (Heneman & Werner, 2004; Heneman, 1992; Ng & Feldman, 2009; Ng & Feldman, 2010).

Finally, a fourth confounding domain we need to consider is so‐called co‐interventions. Relevant co‐interventions are the interventions that individuals might receive after or with the initiation of the intervention of interest, which are related to the intervention received and are prognostic for the outcome of interest (Sterne et al., 2022). Within the scope of this review, relevant co‐interventions are interventions that are somehow related to the application of a merit pay system and may also predict employee work motivation, effort, or performance. We believe related interventions are all compensation practices that determine an employee's salary increase or other salary benefits received beyond the merit increase at a particular time. We already mentioned such an intervention in an earlier paragraph, namely whether the employee just got promoted and may, therefore, have received an additional salary increase or benefits on top of a merit increase (Gerhart, 2023). Other relevant co‐interventions are whether merit increases are combined with other types of salary increases, such as market adjustments, cost‐of‐living adjustments, or seniority increases, or with other P4P plans, such as awarding merit bonuses or group‐based incentive plans (Gerhart, 2023; Harris et al., 1998; Nyberg et al., 2016).

For each of the previously discussed confounders, we will assess whether they were appropriately measured and controlled for. Furthermore, we will assess whether and how additional confounding domains or co‐interventions were addressed, relevant to the setting of that particular study, or which the study authors identified as important.

#### 3.3.5 Measures of treatment effect

If a study reports both adjusted and unadjusted effects, we will extract both. Yet, considering (nearly) all the studies will be non‐randomized studies of intervention effects, we follow the recommendation that adjusted effects (i.e., estimates based on analysis that attempt to control for confounding) should be preferred over unadjusted effects if both are reported (Reeves et al., 2022). If multiple (adjusted) estimates are reported for the same outcome in a study, we will choose the one that minimizes the risk of bias due to confounding the most. Referring to the common design features of studies on the outcomes of merit pay, the partial effect sizes (i.e., adjusted effects) we most often expect to encounter are regression coefficiënts. Other studies may only report bivariate effect sizes such as (un)standardized mean differences or correlations.

We will use standard calculation procedures to calculate the effect size which we will consider most appropriate for our analysis and report what formulas were used. When studies do not report the most desirable statistics for calculating the selected effect size, we will contact the authors to see if they can provide them, consult sources for alternative approaches to calculate them (e.g., Aloe & Thompson, 2013), or consult the Campbell methodologists on the approach to follow.

#### 3.3.6 Unit of analysis issues

We focus on individual‐level studies to assess whether merit pay predicts future employee work motivation, effort, and performance. As mentioned above, we will use the quasi‐experimental taxonomy features checklist to describe each eligible study (Reeves et al., 2017). This checklist includes several questions related to clustering and can, therefore, uncover issues related to the unit of analysis. One form of ‘implicit’ clustering that may occur in studies on merit pay is that merit increases are often allocated based on performance ratings by the employee's direct supervisor. As a consequence, if two or more employees included in the data set were rated by the same supervisor, then they form a cluster. Moreover, one of our outcomes of interest, employee work performance, is also often measured through supervisor ratings, which again creates clustered data if managers rate multiple employees. The extent to which supervisors rate their employees based, in part, on their own idiosyncratic tendencies and/or biases, makes any of the employees’ merit increases or performance ratings partially dependent on their supervisor. Although this dependency which can likely attenuate effect sizes is frequently acknowledged, it is often ignored in individual‐level research in organizational psychology and organizational behavior (Bliese & Hanges, 2004; Ellington et al., 2021). The same seems to apply to research on merit pay. So far, we have found only two studies on merit pay that handled this issue (Nyberg et al., 2016; Park & Sturman, 2016). We will explore potential ways effect sizes can be adjusted for this dependency (e.g., Ellington et al., 2021) and consult the Campbell methodologists on how this issue can best be addressed in the review.

We also expect that different forms of so‐called multiplicity in effect sizes may cause statistical dependency in our meta‐analytic data set. First of all, some studies might report effect sizes for different measures of our outcome of interest (e.g., effect sizes for 2 or more dimensions of employee work performance or different types of motivation are reported). Second, some studies might measure our outcomes of interest over multiple time points. How we will address these instances of multiplicity, depends on factors that we will only be able to oversee once we have collected all the eligible studies, for example, the degree to which these effect sizes are interchangeable or equivalent. We will use the decision tree suggested by López‐López and his colleagues (López‐López et al., 2018) to determine the most appropriate approach for dealing with any multiplicity encountered, for example, using a multilevel model, averaging the effect sizes, or performing separate meta‐analyses.

Finally, we will also assess whether there might be any form of between‐study dependency among the eligible studies (e.g., studies performed in the same country) that should be accounted for and addressed (i.e., using a multilevel model) (Gooty et al., 2021; Konstantopoulos, 2011).

#### 3.3.7 Criteria for determination of independent findings

Depency issues issues were discussed together with unit of analysis issues in the previous section (see 3.3.6).

#### 3.3.8 Dealing with missing data

If data seems to be missing from a report, we will reach out to the authors. Alternatively, we will search for other reports on the study or consult the Campbell methodologist regarding permissible approaches to deal with the missing pieces of information.

#### 3.3.9 Assessment of heterogeneity

Considering most, if not all, included studies will be non‐randomized, greater heterogeneity should be expected (Reeves et al., 2022). Indeed, we expect that the variation in effect sizes across studies may depend on the type of study design, how the outcomes were measured (e.g., subjective vs. objective performance ratings), the type of effect size (i.e., partial vs. bivariate), what controls or covariates were included in the analysis, and other factors related to the different domains of bias that will be coded for each study. To evaluate for each outcome category whether there is heterogeneity in the effect sizes, we will report Q and perform the chi‐squared statistical test for heterogeneity, as it assesses whether observed differences in results are compatible with chance alone (Deeks et al., 2022). Since this test might be underpowered by a small number of studies, we will also report and interpret *T*² and *I*² (Borenstein, 2019). If possible, the results will be displayed in a forest plot sorted by outcome and study design feature or risk of bias. We will also calculate and report the prediction interval to evaluate the amount of heterogeneity and its implications. Given the numerous factors listed on which the variation in effect sizes may depend, we will, as recommended (Borenstein, 2019), be particularly careful in interpreting the heterogeneity statistics when we count only a small number (i.e., <10) of studies for the outcome in question.

#### 3.3.10 Assessment of reporting biases

We presume that non‐reporting biases might be an issue in the research on merit pay in that studies with nonsignificant findings are less likely to be published (i.e., publication bias) or those outcomes with nonsignificant results are omitted (i.e., selective reporting). To assess and appraise their potential for distorting the results of the review, we will follow the recommendations of the corresponding chapter in the Cochrane Handbook for Systematic Reviews of Interventions (Page et al., 2022). Specifically, to assess publication bias we will generate a funnel plot and visually inspect its symmetry. If the number of studies is sufficient (i.e., >10), we will also statistically test the funnel plot asymmetry. When there is evidence of funnel plot asymmetry, we will consider to which degree other sources than non‐reporting bias may explain this asymmetry (e.g., poor methodological quality, true heterogeneity). Given the limitations of funnel plot methods, we also plan to reanalyze the data with a selection model (Maier et al., 2022) or to adopt another approach when that seems appropriate, in consultation with the Campbell methodologists.

#### 3.3.11 Data synthesis

We will perform separate meta‐analyses for our three outcomes of interest. Depending on the nature and degree of dependency within and between the eligible studies, applying a multilevel model might be most adequate. We plan to incorporate both bivariate and partial effects into the same meta‐analysis, but given that they estimate different parameters we will use sensitivity analyses and/or moderator analyses to model the differences between them. We might eventually synthesize the bivariate and partial effect sizes in separate meta‐analyses if that seems more appropriate (Aloe et al., 2016). Since we expect the covariates included in the regression model will vary from study to study, we will choose, if feasible, a method of synthesis that can deal with regression coefficients from different regression models in consultation with experts on the matter (e.g., Fernández‐Castilla et al., 2019). We will also apply the inverse‐variance method so that larger studies with smaller standard errors will be given more weight than smaller studies with larger standard errors, which will minimize the imprecision of our pooled effect estimates (Deeks et al., 2022). Given the many ways in which eligible studies may differ from one another, including sample and intervention characteristics, applying a random‐effects model is most appropriate (Borenstein et al., 2021; Reeves et al., 2022). The software that we will use for storing and analyzing the data will be RevMan and R. Summary estimates will only be reported if the features of (a subgroup of) the studies can be considered sufficiently similar to be combined in a meta‐analysis. If not sufficient and consistent effect estimates are available or calculable to be pooled, we will provide a structured summary (i.e., tabulation) of the effects for each study, organized by our risk of bias judgments so that more trustworthy evidence is more prominently visible. (McKenzie & Brennan, 2022).

#### 3.3.12 Subgroup analysis and investigation of heterogeneity

As discussed in the background section of this protocol, we expect that the association between merit pay and subsequent employee work motivation, effort, and performance, might be moderated by the actual and the perceived relationship between performance and merit pay. Since both are continuous variables, we suggest performing a meta‐regression for each outcome to test whether they are associated with the effect sizes in the studies. Since we expect that the heterogeneity is also attributable to other study differences, a random‐effects model will be applied (Borenstein et al., 2021). As for motivation, we expect its association with merit pay will be moderated by the type of work motivation that was measured (i.e., general, intrinsic, or extrinsic). Since motivation type is a categorical variable, we will use the *Q*‐test while applying a random effects model to assess the relationship between this variable and the effect size. We will use the same approach for other categorical variables that we presume may also partially explain the variation in effect sizes across studies, namely the type of study design, our risk of bias judgments, and what type of outcome measure was used. Finally, we might also test some additional moderators, for instance, sector or cultural region, and present them as exploratory post‐hoc analysis. We recognize that the appropriateness of subgroup analyses and meta‐regressions depends on the number of studies. If the number of studies appears insufficient to assess specific moderators for a specific outcome, we will discuss with Campbell methodologists what approach to adopt.

#### 3.3.13 Sensitivity analysis

As explained earlier, we might have to make some judgment calls on how to deal with dependency or multiplicity in effect sizes. We will repeat the concerned meta‐analyses to verify whether the findings would not have been different had we made alternative choices. Of course, other issues suitable for sensitivity analysis may arise during the review process and will be reported and treated accordingly.

#### 3.3.14 Treatment of qualitative research

We do not plan to include qualitative research.

#### 3.3.15 Summary of findings and assessment of the certainty of the evidence

We do not plan to include a “Summary of findings and assessment of the certainty of the evidence.”

## CONTRIBUTIONS OF AUTHORS

- Content: Cédric Velghe and Dr. Stan De Spiegelaere—Cédric Velghe is a work and organizational psychologist and also gained relevant content expertise through his involvement in advisory projects on employee compensation for private and public organizations. Dr. Stan De Spiegelaere has obtained a master in labor sciences and a PhD in sociology. He has gained relevant content expertise through his research and his affiliation as a guest professor at Ghent University and research director at UNI Europa.
- Systematic review methods: Cédric Velghe, Anders McIlquham‐Schmidt, Dr. Pinar Celik & Dr. Martin Storme—Cédric Velghe and Anders McIlquham‐Schmidt have developed expertise in systematic review methods by completing numerous Rapid Evidence Assessments and Critically Appraised Topics for advisory purposes. Dr. Pinar Celik and Dr. Martin Storme have published a systematic review and meta‐analyses.
- Statistical analysis: Cédric Velghe and Dr. Martin Storme—Finally, Cédric Velghe has developed skills in statistical analyses through several R&D projects and data‐analytical projects. Dr. Dr. Martin Storme performed the statistical analysis for several meta‐analyses, and also developed expertise in this field through his other research projects.
- Information retrieval: Cédric Velghe developed the search strategy in consultation with an information expert and will perform the electronic searches and gray literature searches.

## DECLARATIONS OF INTEREST

There are no potential conflicts of interest to declare.

## PRELIMINARY TIMEFRAME

Our approximate date for submission of the systematic review is within 1,5 years of protocol approval.

## PLANS FOR UPDATING THIS REVIEW

The lead author, Cédric Velghe, intends to organize an update of this review every 5 years, based on the availability of new research.

## DATA AND ANALYTIC CODE

We will submit supplementary material including data coding sheets and analytic codes.

## SOURCES OF SUPPORT

### Internal sources

No sources of support provided.

### External sources

No sources of support provided.

## Supporting information

Supporting information.

Supporting information.

Supporting information.

## Data Availability

We will submit supplementary material including data coding sheets and analytic codes.
