# Supplementary material for: PROTOCOL: Employee work motivation, effort, and performance under a merit pay system: A systematic review
Source: Campbell Syst Rev. 2024 Oct 30;20(4):e70001. doi: 10.1002/cl2.70001 (PMC11522831; doi:10.1002/cl2.70001)
Supplement: Supplementary file 1 — Supporting information. [file CL2-20-e70001-s003.pdf]

Appendix 1 - Search strategies

ABI/Inform Collection - Search strategy

The database, ABI/INFORM Collection, will be accessed through ProQuest.

Overview of the search strategy

Concepts	Related terms
Intervention	Subject headings: "Merit increases", "Raises", "Pay for performance"<br><br>Keywords: "merit pay", “merit raise*”, "merit increase*", "pay raise*", "pay increase*", "salary raise*", "salary increase*", "wage increase*"
Outcomes	Subject headings: motivation, "labor productivity", "Organizational behavior", "Human performance"<br><br>Keywords: motivation, effort, performance, productiv*

Applied limiting commands

- Exclude wire feeds
- Source type:
  - Include:
    - Scholarly Journals
    - Dissertations & Theses
    - Conference Papers & Proceedings
    - Working Papers
  - Exclude:
    - Trade Journals
    - Newspapers
    - Reports
    - Magazines
    - Blogs, Podcasts, & Websites
    - Other Sources
    - Books

Proposed search strategy

Search date: 14th of August 2024
Name of searcher: Cédric Velghe
Export ID: 1.1

#	**Searches**	**Results**
1	MAINSUBJECT.EXACT("Raises") OR MAINSUBJECT.EXACT("Merit increases") OR MAINSUBJECT.EXACT("Pay	2.867

#	Searches	Results
	for performance")	
2	title("merit pay" OR “merit raise*” OR "merit increase*" OR "pay raise*" OR "pay increase*" OR "salary raise*" OR "salary increase*" OR "wage increase*")	350
3	abstract("merit pay" OR “merit raise*” OR "merit increase*" OR "pay raise*" OR "pay increase*" OR "salary raise*" OR "salary increase*" OR "wage increase*")	3.127
4	1 OR 2 OR 3 MAINSUBJECT.EXACT("Raises") OR MAINSUBJECT.EXACT("Merit increases") OR MAINSUBJECT.EXACT("Pay for performance") OR title("merit pay" OR “merit raise*” OR "merit increase*" OR "pay raise*" OR "pay increase*" OR "salary raise*" OR "salary increase*" OR "wage increase*") OR abstract("merit pay" OR “merit raise*” OR "merit increase*" OR "pay raise*" OR "pay increase*" OR "salary raise*" OR "salary increase*" OR "wage increase*")	5.778
5	MAINSUBJECT.EXACT("Labor productivity") OR MAINSUBJECT.EXACT("Motivation") OR MAINSUBJECT.EXACT("Organizational behavior") OR MAINSUBJECT.EXACT("Human performance")	77.036
6	title(motivation OR effort OR performance OR productiv*)	145.255
7	abstract(motivation OR effort OR performance OR productiv*)	646.539
8	5 OR 6 OR 7 MAINSUBJECT.EXACT("Labor productivity") OR MAINSUBJECT.EXACT("Motivation") OR MAINSUBJECT.EXACT("Organizational behavior") OR MAINSUBJECT.EXACT("Huma	712.085

#	Searches	Results
	n performance") OR title(motivation OR effort OR performance OR productiv*) OR abstract(motivation OR effort OR performance OR productiv*)	
9	4 AND 8 (MAINSUBJECT.EXACT("Raises") OR MAINSUBJECT.EXACT("Merit increases") OR MAINSUBJECT.EXACT("Pay for performance") OR title("merit pay" OR “merit raise*” OR "merit increase*" OR "pay raise*" OR "pay increase*" OR "salary raise*" OR "salary increase*" OR "wage increase*") OR abstract("merit pay" OR “merit raise*” OR "merit increase*" OR "pay raise*" OR "pay increase*" OR "salary raise*" OR "salary increase*" OR "wage increase*"))AND (MAINSUBJECT.EXACT("Labor productivity") OR MAINSUBJECT.EXACT("Motivation") OR MAINSUBJECT.EXACT("Organizational behavior") OR MAINSUBJECT.EXACT("Human performance") OR title(motivation OR effort OR performance OR productiv*) OR abstract(motivation OR effort OR performance OR productiv*))	3.018

Social Science Premium Collection - Search strategy

The database, Social Science Premium Collection, will be accessed through ProQuest.

Overview of the search strategy

Concepts	Related terms
Intervention	Subject headings: "Merit increases", "Raises", "Pay for performance"<br><br>Keywords: "merit pay", “merit raise*”, "merit increase*", "pay raise*", "pay increase*", "salary

	raise*", "salary increase*", "wage increase*"
Outcomes	Subject headings: Motivation, "Labor productivity", "Organizational behavior", "Human performance"<br><br>Keywords: motivation, effort, performance, productiv*

Applied limiting commands

- Source type:
  - Include:
    - Scholarly Journals
    - Dissertations & Theses
    - Conference Papers & Proceedings
    - Working Papers
  - Exclude:
    - Trade Journals
    - Newspapers
    - Reports
    - Magazines
    - Blogs, Podcasts, & Websites
    - Other Sources
    - Wire feeds

Proposed search strategy

Search date: 15th of August 2024
Name of searcher: Cédric Velghe
Export ID: 11.1

#	**Searches**	**Results**
1	MAINSUBJECT.EXACT("Raises") OR MAINSUBJECT.EXACT("Merit increases") OR MAINSUBJECT.EXACT("Pay for performance")	1.776
2	title("merit pay" OR "merit raise*" OR "merit increase*" OR "pay raise*" OR "pay increase*" OR "salary raise*" OR "salary increase*" OR "wage increase*")	1.125
3	abstract("merit pay" OR "merit raise*" OR "merit increase*" OR "pay raise*" OR "pay increase*" OR "salary raise*" OR "salary increase*" OR "wage increase*")	4.153
4	1 OR 2 OR 3 MAINSUBJECT.EXACT("Raises") OR MAINSUBJECT.EXACT("Merit	6.353

#	Searches	Results
	increases") OR MAINSUBJECT.EXACT("Pay for performance") OR title("merit pay" OR "merit raise*" OR "merit increase*" OR "pay raise*" OR "pay increase*" OR "salary raise*" OR "salary increase*" OR "wage increase*") OR abstract("merit pay" OR "merit raise*" OR "merit increase*" OR "pay raise*" OR "pay increase*" OR "salary raise*" OR "salary increase*" OR "wage increase*")	
5	MAINSUBJECT.EXACT("Labor productivity") OR MAINSUBJECT.EXACT("Motivation") OR MAINSUBJECT.EXACT("Organizational behavior") OR MAINSUBJECT.EXACT("Human performance")	122.851
6	title(motivation OR effort OR performance OR productiv*)	238.381
7	abstract(motivation OR effort OR performance OR productiv*)	1.238.063
8	5 OR 6 OR 7 MAINSUBJECT.EXACT("Labor productivity") OR MAINSUBJECT.EXACT("Motivation") OR MAINSUBJECT.EXACT("Organizational behavior") OR MAINSUBJECT.EXACT("Human performance") OR title(motivation OR effort OR performance OR productiv*) OR abstract(motivation OR effort OR performance OR productiv*)	1.361.819
9	4 AND 8 (MAINSUBJECT.EXACT("Raises") OR MAINSUBJECT.EXACT("Merit increases") OR MAINSUBJECT.EXACT("Pay for performance") OR title("merit pay" OR "merit raise*" OR "merit increase*" OR "pay raise*" OR "pay increase*" OR "salary raise*"	2.597

#	Searches	Results
	OR "salary increase*" OR "wage increase*") OR abstract("merit pay" OR "merit raise*" OR "merit increase*" OR "pay raise*" OR "pay increase*" OR "salary raise*" OR "salary increase*" OR "wage increase*")) AND (MAINSUBJECT.EXACT("Labor productivity") OR MAINSUBJECT.EXACT("Motivation") OR MAINSUBJECT.EXACT("Organizational behavior") OR MAINSUBJECT.EXACT("Human performance") OR title(motivation OR effort OR performance OR productiv*) OR abstract(motivation OR effort OR performance OR productiv*))	

Web of Science - Search strategy

The databases, Web of Science Core Collection, KCI-Koran Journal Database, and the SciELO Citation Index, will be accessed separately through Web of Science.

Overview of the search strategy

Concepts	Related terms
Intervention	Subject headings: Not available<br><br>Keywords: "merit pay", “merit raise*”, "merit increase*", "pay raise*", "pay increase*", "salary raise*", "salary increase*", "wage increase*"
Outcomes	Subject headings: Not available<br><br>Keywords: motivation, effort, performance, productiv*

Applied limiting commands for Web of Science Core Collection

- Document Types:
    - Include:
        - Article
        - Proceeding Paper
        - Early Access
        - Review Article
        - Meeting Abstract
        - Correction

        - Retracted Publication
    - Exclude:
        - Editorial Material
        - Letter
        - News item
        - Note
        - Book Review
        - Book Chapters

Proposed search strategy for Web of Science Core Collection

Search date: 14th of August 2024
Name of searcher: Cédric Velghe
Export ID: 5.1

#	Searches	Results
1	TS = ("merit pay" OR "merit raise*" OR "merit increase*" OR "pay raise*" OR "pay increase*" OR "salary raise*" OR "salary increase*" OR "wage increase*")	2.290
2	TS = (motivation OR effort OR performance OR productiv*)	
3	1 AND 2<br>TS = (("merit pay" OR "merit raise*" OR "merit increase*" OR "pay raise*" OR "pay increase*" OR "salary raise*" OR "salary increase*" OR "wage increase*") AND (motivation OR effort OR performance OR productiv*))	810

Applied limiting commands for KCI-Korean Journal Database

- None

Proposed search strategy for KCI-Korean Journal Database

Search date: 14th of August 2024
Name of searcher: Cédric Velghe
Export ID: 5.2

#	Searches	Results
1	TS = ("merit pay" OR "merit raise*" OR "merit increase*" OR "pay raise*" OR "pay increase*" OR "salary raise*" OR "salary increase*" OR "wage increase*")	451
2	TS = (motivation OR effort OR performance OR productiv*)	284.859
3	1 AND 2	152

#	Searches	Results
	TS = (("merit pay" OR "merit raise*" OR "merit increase*" OR "pay raise*" OR "pay increase*" OR "salary raise*" OR "salary increase*" OR "wage increase*") AND (motivation OR effort OR performance OR productiv*))	

Applied limiting commands for SciELO Citation Index

- Document Types:
    - Include:
        - Research Article
    - Exclude:
        - Book Review
        - Editorial

Proposed search strategy for SciELO Citation Index

Search date: 14th of August 2024
Name of searcher: Cédric Velghe
Export ID: 5.3

#	Searches	Results
1	TS = ("merit pay" OR "merit raise*" OR "merit increase*" OR "pay raise*" OR "pay increase*" OR "salary raise*" OR "salary increase*" OR "wage increase*")	54
2	TS = (motivation OR effort OR performance OR productiv*)	93.698
3	1 AND 2<br>TS = (("merit pay" OR "merit raise*" OR "merit increase*" OR "pay raise*" OR "pay increase*" OR "salary raise*" OR "salary increase*" OR "wage increase*") AND (motivation OR effort OR performance OR productiv*))	11

Directory of Open Access Journals - Search strategy

The database, Directory of Open Access Journals, will be accessed.

Overview of the search strategy

Concepts	Related terms
Intervention	Subject headings: Not available

	Keywords: "merit pay", “merit raise*”, "merit increase*", "pay raise*", "pay increase*", "salary raise*", "salary increase*", "wage increase*"
Outcomes	Subject headings: Not available<br><br>Keywords: motivation, effort, performance, productiv*

Applied limiting commands

- None

Proposed search strategy

Search date: 14th of August 2024
Name of searcher: Cédric Velghe
Export ID: 6.1

#	Searches	Results
1	All fields ("merit pay")	30
2	All fields ("merit raise*")	0
3	All fields ("merit increase*")	4
4	All fields ("pay raise*")	8
5	All fields ("pay increase*")	5
6	All fields ("salary raise*")	1
7	All fields ("salary increase*")	35
8	All fields ("wage increase*")	59
9	All fields (motivation)	60.190
10	All fields (effort)	68.686
11	All fields (performance)	742.506
12	All fields (productiv*)	135.829
13	1 AND 9<br>All fields ("merit pay" motivation)	4
14	1 AND 10<br>All fields ("merit pay" effort)	0
15	1 AND 11<br>All fields ("merit pay" performance)	16

#	Searches	Results
16	1 AND 12<br>All fields ("merit pay" productiv*)	1
17	2 AND 9<br>All fields ("merit raise*" motivation)	0
18	2 AND 10<br>All fields ("merit raise*" effort)	0
19	2 AND 11<br>All fields ("merit raise*" performance)	0
20	2 AND 12<br>All fields ("merit raise*" productiv*)	0
21	3 AND 9<br>All fields ("merit increase*" motivation)	1
22	3 AND 10<br>All fields ("merit increase*" effort)	0
23	3 AND 11<br>All fields ("merit increase*" performance)	2
24	3 AND 12<br>All fields ("merit increase*" productiv*)	1
25	4 AND 9<br>All fields ("pay raise*" motivation)	0
26	4 AND 10<br>All fields ("pay raise*" effort)	1
27	4 AND 11<br>All fields ("pay raise*" performance)	3
28	4 AND 12<br>All fields ("pay raise*" productiv*)	0
29	5 AND 9<br>All fields ("pay increase*" motivation)	0

#	Searches	Results
30	5 AND 10<br>All fields ("pay increase*" effort)	0
31	5 AND 11<br>All fields ("pay increase*" performance)	1
32	5 AND 12<br>All fields ("pay increase*" productiv*)	0
33	6 AND 9<br>All fields ("salary raise*" motivation)	0
34	6 AND 10<br>All fields ("salary raise*" effort)	0
35	6 AND 11<br>All fields ("salary raise*" performance)	0
36	6 AND 12<br>All fields ("salary raise*" productiv*)	0
37	7 AND 9<br>All fields ("salary increase*" motivation)	6
38	7 AND 10<br>All fields ("salary increase*" effort)	1
39	7 AND 11<br>All fields ("salary increase*" performance)	4
40	7 AND 12<br>All fields ("salary increase*" productiv*)	1
41	8 AND 9<br>All fields ("wage increase*" motivation)	3
42	8 AND 10<br>All fields ("wage increase*" effort)	1
43	8 AND 11<br>All fields ("wage increase*"	2

#	Searches	Results
	performance)	
44	8 AND 12<br>All fields ("wage increase*" productiv*)	7

China Journals, Doctoral Dissertations & Master's Theses, and Proceedings of Conference - Search strategy

The databases, China Academic Journals, Doctoral Dissertations & Master's Theses, and Proceedings of Conference, will be accessed via CNKI.

Overview of the search strategy

Concepts	Related terms
Intervention	Subject headings: Not available<br><br>Keywords: "merit pay", “merit raise”, "merit increase", "pay raise", "pay increase", "salary raise", "salary increase", "wage increase"
Outcomes	Subject headings: Not available<br><br>Keywords: motivation, effort, performance, productivity, productiveness

Note: CNKI does not seem to use wildcards. Using a keyword in its singular form also seems to catch its plural.

Applied limiting commands

- Cross-Language Search unchecked
- Include:
  - Academic Journal
  - Theses & Dissertations
  - Conferences
- Exclude:
  - Newspapers
  - Yearbooks
  - Books
  - Patents
  - Standards
  - Achievements

Proposed search strategy for

Search date: 14th of August 2024
Name of searcher: Cédric Velghe
Export ID: 7.1

#	Searches	Results
1	(Title, Keyword and abstract: "merit pay" + "merit raise" + "merit increase" + "pay raise" + "pay increase" + "salary raise" + "salary increase") OR (Title, Keyword and abstract: "wage increase") *(Note: A new search line was added with OR, because CNKI limits the number of characters that can be added in one search field)*	3.312
2	(Title, Keyword and abstract: motivation + effort + performance + productivity + productiveness)	3.731.201
3	1 AND 2 (Title, Keyword and abstract: "merit pay" + "merit raise" + "merit increase" + "pay raise" + "pay increase" + "salary raise" + "salary increase") OR (Title, Keyword and abstract: "wage increase") AND (Title, Keyword and abstract: motivation + effort + performance + productivity + productiveness)	1.340

Research Papers in Economics - Search strategy

The database, Research Papers in Economics (RePEc), will be accessed via EconPapers.

Overview of the search strategy

Concepts	Related terms
Intervention	Subject headings: Not available<br><br>Keywords: "merit pay", “merit raise*”, "merit increase*", "pay raise*", "pay increase*", "salary raise*", "salary increase*", "wage increase*"
Outcomes	Subject headings: Not available<br><br>Keywords: motivation, effort, performance, productiv*

Applied limiting commands

- Checked:
    - Working papers
    - Journal Articles
- Unchecked:

- Books & Chapters
- Software items
- Registered authors
- Online Items Only

Proposed search strategy

Search date: 15th of August 2024
Name of searcher: Cédric Velghe
Export ID: 8.1

#	Searches	Results
1	Free text search: ("merit pay" OR "merit raise*" OR "merit increase*" OR "pay raise*" OR "pay increase*" OR "salary raise*" OR "salary increase*" OR "wage increase*")	3.020
2	Free text search: (motivation OR effort OR performance OR productiv*)	633.789
3	1 AND 2<br>Free text search: ("merit pay" OR "merit raise*" OR "merit increase*" OR "pay raise*" OR "pay increase*" OR "salary raise*" OR "salary increase*" OR "wage increase*") AND (motivation OR effort OR performance OR productiv*)	834

Social Science Research Network - Search strategy

The database, Social Science Research Network (SSRN), will be accessed.

Overview of the search strategy

Concepts	Related terms
Intervention	Subject headings: Not available<br><br>Keywords: "merit pay", “merit raise*”, "merit increase*", "pay raise*", "pay increase*", "salary raise*", "salary increase*", "wage increase*"
Outcomes	Subject headings: Not available<br><br>Keywords: motivation, effort, performance, productiv*

Applied limiting commands

- None

Proposed search strategy

Search date: 15th of August 2024
Name of searcher: Cédric Velghe
Export ID: 9.1-7

#	Searches	Results
1	Title, Abstract & Keywords: "merit pay"	32
2	Title, Abstract & Keywords: "merit raise"	2
3	Title, Abstract & Keywords: "merit raises"	2
4	Title, Abstract & Keywords: "merit increase"	2
5	Title, Abstract & Keywords: "merit increases"	2
6	Title, Abstract & Keywords: "pay raise"	13
7	Title, Abstract & Keywords: "pay raises"	24
8	Title, Abstract & Keywords: "pay increase"	29
9	Title, Abstract & Keywords: "pay increases"	87
10	Title, Abstract & Keywords: "salary raise"	2
11	Title, Abstract & Keywords: "salary raises"	3
12	Title, Abstract & Keywords: "salary increase"	36
13	Title, Abstract & Keywords: "salary increases"	56
14	Title, Abstract & Keywords: "wage increase"	229
15	Title, Abstract & Keywords: "wage increases"	528
16	Title, Abstract & Keywords: motivation	10.812
17	Title, Abstract & Keywords: effort	26.004
18	Title, Abstract & Keywords: performance	157.015
19	Title, Abstract & Keywords: productivity	31.259
20	Title, Abstract & Keywords: productiveness	24
21	1 AND 16	1

#	Searches	Results
	Title, Abstract & Keywords: "merit pay" motivation	
22	1 AND 17<br>Title, Abstract & Keywords: "merit pay" effort	7
23	1 AND 18<br>Title, Abstract & Keywords: "merit pay" performance	18
24	1 AND 19<br>Title, Abstract & Keywords: "merit pay" productivity	4
25	1 AND 20<br>Title, Abstract & Keywords: "merit pay" productiveness	0
26	2 AND 16<br>Title, Abstract & Keywords: "merit raise" motivation	0
27	2 AND 17<br>Title, Abstract & Keywords: "merit raise" effort	0
28	2 AND 18<br>Title, Abstract & Keywords: "merit raise" performance	1
29	2 AND 19<br>Title, Abstract & Keywords: "merit raise" productivity	0
30	2 AND 20<br>Title, Abstract & Keywords: "merit raise" productiveness	0
31	3 AND 16<br>Title, Abstract & Keywords: "merit raises" motivation	0
32	3 AND 17<br>Title, Abstract & Keywords: "merit raises" effort	1
33	3 AND 18<br>Title, Abstract & Keywords: "merit raises" performance	2
34	3 AND 19<br>Title, Abstract & Keywords: "merit raises" productivity	0

#	Searches	Results
35	3 AND 20<br>Title, Abstract & Keywords:<br>"merit raises" productiveness	0
36	4 AND 16<br>Title, Abstract & Keywords:<br>"merit increase" motivation	0
37	4 AND 17<br>Title, Abstract & Keywords:<br>"merit increase" effort	0
38	4 AND 18<br>Title, Abstract & Keywords:<br>"merit increase" performance	1
39	4 AND 19<br>Title, Abstract & Keywords:<br>"merit increase" productivity	1
40	4 AND 20<br>Title, Abstract & Keywords:<br>"merit increase"<br>productiveness	0
41	5 AND 16<br>Title, Abstract & Keywords:<br>"merit increases" motivation	0
42	5 AND 17<br>Title, Abstract & Keywords:<br>"merit increases" effort	0
43	5 AND 18<br>Title, Abstract & Keywords:<br>"merit increases"<br>performance	1
44	5 AND 19<br>Title, Abstract & Keywords:<br>"merit increases" productivity	0
45	5 AND 20<br>Title, Abstract & Keywords:<br>"merit increases"<br>productiveness	0
46	6 AND 16<br>Title, Abstract & Keywords:<br>"pay raise" motivation	2
47	6 AND 17<br>Title, Abstract & Keywords:<br>"pay raise" effort	2

#	Searches	Results
48	6 AND 18<br>Title, Abstract & Keywords:<br>"pay raise" performance	2
49	6 AND 19<br>Title, Abstract & Keywords:<br>"pay raise" productivity	2
50	6 AND 20<br>Title, Abstract & Keywords:<br>"pay raise" productiveness	0
51	7 AND 16<br>Title, Abstract & Keywords:<br>"pay raises" motivation	0
52	7 AND 17<br>Title, Abstract & Keywords:<br>"pay raises" effort	4
53	7 AND 18<br>Title, Abstract & Keywords:<br>"pay raises" performance	9
54	7 AND 19<br>Title, Abstract & Keywords:<br>"pay raises" productivity	7
55	7 AND 20<br>Title, Abstract & Keywords:<br>"pay raises" productiveness	0
56	8 AND 16<br>Title, Abstract & Keywords:<br>"pay increase" motivation	1
57	8 AND 17<br>Title, Abstract & Keywords:<br>"pay increase" effort	1
58	8 AND 18<br>Title, Abstract & Keywords:<br>"pay increase" performance	10
59	8 AND 19<br>Title, Abstract & Keywords:<br>"pay increase" productivity	3
60	8 AND 20<br>Title, Abstract & Keywords:<br>"pay increase"<br>productiveness	0
61	9 AND 16	1

#	Searches	Results
	Title, Abstract & Keywords: "pay increases" motivation	
62	9 AND 17<br>Title, Abstract & Keywords: "pay increases" effort	8
63	9 AND 18<br>Title, Abstract & Keywords: "pay increases" performance	26
64	9 AND 19<br>Title, Abstract & Keywords: "pay increases" productivity	14
65	9 AND 20<br>Title, Abstract & Keywords: "pay increases" productiveness	0
66	10 AND 16<br>Title, Abstract & Keywords: "salary raise" motivation	0
67	10 AND 17<br>Title, Abstract & Keywords: "salary raise" effort	0
68	10 AND 18<br>Title, Abstract & Keywords: "salary raise" performance	1
69	10 AND 19<br>Title, Abstract & Keywords: "salary raise" productivity	0
70	10 AND 20<br>Title, Abstract & Keywords: "salary raise" productiveness	0
71	11 AND 16<br>Title, Abstract & Keywords: "salary raises" motivation	0
72	11 AND 17<br>Title, Abstract & Keywords: "salary raises" effort	0
73	11 AND 18<br>Title, Abstract & Keywords: "salary raises" performance	1
74	11 AND 19<br>Title, Abstract & Keywords:	0

#	Searches	Results
	"salary raises" productivity	
75	11 AND 20<br>Title, Abstract & Keywords: "salary raises" productiveness	0
76	12 AND 16<br>Title, Abstract & Keywords: "salary increase" motivation	1
77	12 AND 17<br>Title, Abstract & Keywords: "salary increase" effort	3
78	12 AND 18<br>Title, Abstract & Keywords: "salary increase" performance	9
79	12 AND 19<br>Title, Abstract & Keywords: "salary increase" productivity	4
80	12 AND 20<br>Title, Abstract & Keywords: "salary increase" productiveness	0
81	13 AND 16<br>Title, Abstract & Keywords: "salary increases" motivation	2
82	13 AND 17<br>Title, Abstract & Keywords: "salary increases" effort	2
83	13 AND 18<br>Title, Abstract & Keywords: "salary increases" performance	12
84	13 AND 19<br>Title, Abstract & Keywords: "salary increases" productivity	5
85	13 AND 20<br>Title, Abstract & Keywords: "salary increases" productiveness	0
86	14 AND 16<br>Title, Abstract & Keywords:	3

#	Searches	Results
	"wage increase" motivation	
87	14 AND 17<br>Title, Abstract & Keywords:<br>"wage increase" effort	12
88	14 AND 18<br>Title, Abstract & Keywords:<br>"wage increase" performance	16
89	14 AND 19<br>Title, Abstract & Keywords:<br>"wage increase" productivity	15
90	14 AND 20<br>Title, Abstract & Keywords:<br>"wage increase" productiveness	0
91	15 AND 16<br>Title, Abstract & Keywords:<br>"wage increases" motivation	3
92	15 AND 17<br>Title, Abstract & Keywords:<br>"wage increases" effort	26
93	15 AND 18<br>Title, Abstract & Keywords:<br>"wage increases" performance	37
94	15 AND 19<br>Title, Abstract & Keywords:<br>"wage increases" productivity	65
95	15 AND 20<br>Title, Abstract & Keywords:<br>"wage increases" productiveness	0

CAIRN - Search strategy

The database, CAIRN, will be accessed.

Overview of the search strategy

Concepts	Related terms
Intervention	Subject headings: Not available<br><br>Keywords: "rémunération au mérite", "salaire au mérite"; "augmentation* au mérite",

	"augmentation* d* mérite", "augmentation* d* salaire*", "augmentation* salar*", "augmentation* de la rémunération", "hausse* d* salaire*", "rémunération à la performance", "augmentation* à la performance", augmentation* w/7 mérite, rémunération w/5 mérite, augmentation* w/7 performance, rémunération w/5 performance

Applied limiting commands

- None

Proposed search strategy

Search date: 15th of August 2024
Name of searcher: Cédric Velghe
Export ID: 10.1-2

#	Searches	Results
1	Résumé ("rémunération au mérite" OU "salaire au mérite" OU "augmentation* au mérite" OU "augmentation* d* mérite" OU "augmentation* d* salaire*" OU "augmentation* salar*" OU "augmentation* de la rémunération" OU "hausse* d* salaire*" OU "rémunération à la performance" OU "augmentation* à la performance" OU (augmentation* w/7 mérite) OU (rémunération w/5 mérite) OU (augmentation* w/7 performance) OU (rémunération w/5 performance))	109
2	Titre d'article / chapitre ("rémunération au mérite" OU "salaire au mérite" OU "augmentation* au mérite" OU "augmentation* d* mérite" OU "augmentation* d* salaire*" OU "augmentation* salar*" OU "augmentation* de la rémunération" OU "hausse* d* salaire*" OU "rémunération à la performance" OU "augmentation* à la performance" OU (augmentation* w/7 mérite) OU (rémunération w/5 mérite) OU	14

#	Searches	Results
	(augmentation* w/7 performance) OU (rémunération w/5 performance))	

Business Source Complete - Search strategy

The database, Business Source Complete, will be accessed through EBSCOhost.

Overview of the search strategy

Concepts	Related terms
Intervention	Subject headings: "PAY for performance", "WAGE increases"<br><br>Keywords: "merit pay", “merit raise*”, "merit increase*", "pay raise*", "pay increase*", "salary raise*", "salary increase*", "wage increase*"
Outcomes	Subject headings: "EMPLOYEE motivation", "LABOR productivity", "JOB performance", "ORGANIZATIONAL citizenship behavior", "TASK performance"<br><br>Keywords: motivation, effort, performance, productiv*

Applied limiting commands

- Source types:
    - Include:
        - Academic Journals
        - Market Research Reports
    - Exclude:
        - Magazines
        - Trade Publications
        - Newspapers
        - Industry Profiles
        - Country Reports
        - Books
        - SWOT analyses
        - Product Reviews

Proposed search strategy

Search date: 14th of August 2024
Name of searcher: Cédric Velghe
Export ID: 2.1

#	Searches	Results
1	(DE "PAY for performance") OR	3.784

#	Searches	Results
	(DE "WAGE increases")	
2	TI ("merit pay" OR “merit raise*” OR "merit increase*" OR "pay raise*" OR "pay increase*" OR "salary raise*" OR "salary increase*" OR "wage increase*")	327
3	AB ("merit pay" OR “merit raise*” OR "merit increase*" OR "pay raise*" OR "pay increase*" OR "salary raise*" OR "salary increase*" OR "wage increase*")	3.091
4	1 OR 2 OR 3<br>(DE "PAY for performance") OR (DE "WAGE increases") OR TI ("merit pay" OR “merit raise*” OR "merit increase*" OR "pay raise*" OR "pay increase*" OR "salary raise*" OR "salary increase*" OR "wage increase*") OR AB ("merit pay" OR “merit raise*” OR "merit increase*" OR "pay raise*" OR "pay increase*" OR "salary raise*" OR "salary increase*" OR "wage increase*")	5.828
5	(DE "EMPLOYEE motivation") OR (DE "LABOR productivity") OR (DE "JOB performance") OR (DE "ORGANIZATIONAL citizenship behavior") OR (DE "TASK performance")	32.059
6	TI (motivation OR effort OR performance OR productiv*)	141.973
7	AB (motivation OR effort OR performance OR productiv*)	665.867
8	5 OR 6 OR 7<br>(DE "EMPLOYEE motivation") OR (DE "LABOR productivity") OR (DE "JOB performance") OR (DE "ORGANIZATIONAL citizenship behavior") OR (DE "TASK performance") OR TI (motivation OR effort OR performance OR productiv*) OR AB (motivation OR effort OR performance OR productiv*)	695.449
9	4 AND 8<br>((DE "PAY for performance") OR (DE "WAGE increases") OR TI ("merit pay" OR “merit	2.007

#	Searches	Results
	raise*" OR "merit increase*" OR "pay raise*" OR "pay increase*" OR "salary raise*" OR "salary increase*" OR "wage increase*") OR AB ("merit pay" OR "merit raise*" OR "merit increase*" OR "pay raise*" OR "pay increase*" OR "salary raise*" OR "salary increase*" OR "wage increase*")) AND ((DE "EMPLOYEE motivation") OR (DE "LABOR productivity") OR (DE "JOB performance") OR (DE "ORGANIZATIONAL citizenship behavior") OR (DE "TASK performance") OR TI (motivation OR effort OR performance OR productiv*) OR AB (motivation OR effort OR performance OR productiv*))	

APA PsycInfo - Search strategy

The database, APA PsycInfo, will be accessed through EBSCOhost.

Overview of the search strategy

Concepts	Related terms
Intervention	Subject headings: Salaries<br><br>Keywords: "merit pay", "merit raise*", "merit increase*", "pay raise*", "pay increase*", "salary raise*", "salary increase*", "wage increase*"
Outcomes	Subject headings: Motivation, "Employee Motivation", "Extrinsic Motivation", "Intrinsic Motivation", "Energy Expenditure", Performance, "Job Performance", Productivity, "Employee Efficiency", "Employee Productivity", "Organizational Citizenship Behavior"<br><br>Keywords: motivation, effort, performance, productiv*

Applied limiting commands

- Source types:
  - Include:
    - Academic Journals
    - Dissertations

        - Electronic Collections
    - Exclude:
        - Books
        - Encyclopedias

Proposed search strategy

Search date: 9th of April 2023
Name of searcher: Cédric Velghe
Export ID: 3.1

#	Searches	Results
1	DE "Salaries"	4.884
2	TI ("merit pay" OR “merit raise*” OR "merit increase*" OR "pay raise*" OR "pay increase*" OR "salary raise*" OR "salary increase*" OR "wage increase*")	132
3	AB ("merit pay" OR “merit raise*” OR "merit increase*" OR "pay raise*" OR "pay increase*" OR "salary raise*" OR "salary increase*" OR "wage increase*")	651
4	1 OR 2 OR 3<br>DE "Salaries" OR TI ("merit pay" OR “merit raise*” OR "merit increase*" OR "pay raise*" OR "pay increase*" OR "salary raise*" OR "salary increase*" OR "wage increase*") OR AB ("merit pay" OR “merit raise*” OR "merit increase*" OR "pay raise*" OR "pay increase*" OR "salary raise*" OR "salary increase*" OR "wage increase*")	5.273
5	DE "Motivation" OR DE "Employee Motivation" OR DE "Extrinsic Motivation" OR DE "Intrinsic Motivation" OR DE "Employee Efficiency" OR DE "Job Performance" OR DE "Productivity" OR DE "Employee Productivity" OR DE "Performance" OR DE "Energy Expenditure" OR DE "Organizational Citizenship Behavior"	131.988
6	TI (motivation OR effort OR performance OR productiv*)	123.320
7	AB (motivation OR effort OR performance OR productiv*)	616.622

#	Searches	Results
8	5 OR 6 OR 7<br>DE "Motivation" OR DE "Employee Motivation" OR DE "Extrinsic Motivation" OR DE "Intrinsic Motivation" OR DE "Employee Efficiency" OR DE "Job Performance" OR DE "Productivity" OR DE "Employee Productivity" OR DE "Performance" OR DE "Energy Expenditure" OR DE "Organizational Citizenship Behavior" OR TI (motivation OR effort OR performance OR productiv*) OR AB (motivation OR effort OR performance OR productiv*)	687.413
9	4 AND 8<br>(DE "Salaries" OR TI ("merit pay" OR “merit raise*” OR "merit increase*" OR "pay raise*" OR "pay increase*" OR "salary raise*" OR "salary increase*" OR "wage increase*") OR AB ("merit pay" OR “merit raise*” OR "merit increase*" OR "pay raise*" OR "pay increase*" OR "salary raise*" OR "salary increase*" OR "wage increase*")) AND (DE "Motivation" OR DE "Employee Motivation" OR DE "Extrinsic Motivation" OR DE "Intrinsic Motivation" OR DE "Employee Efficiency" OR DE "Job Performance" OR DE "Productivity" OR DE "Employee Productivity" OR DE "Performance" OR DE "Energy Expenditure" OR DE "Organizational Citizenship Behavior" OR TI (motivation OR effort OR performance OR productiv*) OR AB (motivation OR effort OR performance OR productiv*))	1.806

Scopus - Search strategy

The database, Scopus, will be accessed through Elsevier.

Overview of the search strategy

Concepts	Related terms
Intervention	Subject headings: Not available<br><br>Keywords: "merit pay", “merit raise*”, "merit increase*", "pay raise*", "pay increase*", "salary raise*", "salary increase*", "wage increase*"
Outcomes	Subject headings: Not available<br><br>Keywords: motivation, effort, performance, productiv*

Applied limiting commands

- Document type:
  - Include:
    - Article
    - Conference Paper
    - Erratum
    - Retracted
  - Exclude:
    - Review
    - Book Chapter
    - Note
    - Book
    - Letter
    - Short Survey
    - Editorial

Proposed search strategy

Search date: 14th of August 2024
Name of searcher: Cédric Velghe
Export ID: 4.1

#	Searches	Results
1	TITLE-ABS-KEY (*"merit pay"* OR *"merit raise*"*OR *"merit increase*"* OR *"pay raise*"* OR *"pay increase*"* OR *"salary raise*"* OR *"salary increase*"* OR *"wage increase*"*)	3.555
2	TITLE-ABS-KEY (motivation OR effort OR performance OR productiv*)	9.600.882
3	1 AND 2<br><br>TITLE-ABS-KEY (("merit pay" OR "merit raise*" OR "merit increase*" OR "pay raise*" OR "pay increase*" OR "salary raise*" OR "salary increase*"	1.139

#	Searches	Results
	OR "wage increase*") AND (motivation OR effort OR performance OR productiv*))	
