# Supplementary material for: PROTOCOL: Employee work motivation, effort, and performance under a merit pay system: A systematic review
Source: Campbell Syst Rev. 2024 Oct 30;20(4):e70001. doi: 10.1002/cl2.70001 (PMC11522831; doi:10.1002/cl2.70001)
Supplement: Supplementary file 2 — Supporting information. [file CL2-20-e70001-s001.pdf]

Appendix 2 - Screening guide

First-level screening based on title and abstract		
1. Does this document report on a study?		
Yes	Unsure	No - EXCLUDE
2. Is merit pay applied as an intervention in this study?		
Yes	Unsure	No - EXCLUDE
3. Is this study conducted on employees or workers?		
Yes	Unsure	No - EXCLUDE
4. Does this study measure employee work motivation, effort, or performance as an outcome?<br>Instruction: Relevant constructs are (work/task) motivation, intrinsic/extrinsic motivation, behavioral intention, (work/task) effort, (work/task) performance, contextual performance (i.a. OCB, personal initiative, proactive behavior, innovative work behavior), objective performance indicators or outcomes, and productivity.		
Yes	Unsure	No - EXCLUDE
5. Does this study fit the research design requirements?<br>Instruction: Exclude cross-sectional and qualitative designs.		
Yes - INCLUDE	Maybe - INCLUDE	No - EXCLUDE

**Second-level screening based on full text**		
1. Does this document report on a study?		
Yes	Unsure - CONTACT AUTHOR(S)	No - EXCLUDE
2. Is merit pay applied as an intervention in this study?<br>Instruction: The applied intervention should be an employee compensation system in which incremental increases in base salary are provided and where the provision or the size of the increase depends on a rating by the employer of the employee's past performance. Study should report whether or not participants are subject to a merit pay program, whether or not they have received a merit increase, and/or the merit increase size. Exclude merit bonus, i.e. reward involves a one-time raise, aka a lump-sum bonus, that is not then continued as part of the base salary.		
Yes	Unsure - CONTACT AUTHOR(S)	No - EXCLUDE
3. Is this study conducted on employees or workers?<br>Instruction - Relevant populations are part-time or full-time employees or workers of private or public organizations. Exclude non-worker populations (i.a. volunteers, students, job seekers, incapacitated persons, retirees, or self-employed) and workers employed by a third party (i.a. agency workers, leased workers).		
Yes	Unsure - CONTACT AUTHOR(S)	No - EXCLUDE
4. Does this study measure employee motivation, effort, or performance as an outcome?<br>Instruction: Relevant constructs are (work/task) motivation, intrinsic/extrinsic motivation, behavioral intention, (work/task) effort, (work/task) performance, contextual performance (i.a. OCB, personal initiative, proactive behavior, innovative work behavior), objective performance indicators or outcomes, and productivity.		
Yes	Unsure - CONTACT AUTHOR(S)	No - EXCLUDE
5. Does this study fit the research design requirements?<br>Instruction: Exclude cross-sectional and qualitative designs. Include studies when the measurement of the dependent variable(s) happened after the application of merit pay and controls were used: pre-intervention level(s) of dependent variables, base salary level or other confounds, and/or a comparison group. Examples are field experiments, quasi-experiments, before-after studies, longitudinal studies, and case-control studies.		
Yes	Unsure - CONTACT AUTHOR(S)	No - EXCLUDE
