# Supplementary material for: PROTOCOL: Employee work motivation, effort, and performance under a merit pay system: A systematic review
Source: Campbell Syst Rev. 2024 Oct 30;20(4):e70001. doi: 10.1002/cl2.70001 (PMC11522831; doi:10.1002/cl2.70001)
Supplement: Supplementary file 3 — Supporting information. [file CL2-20-e70001-s002.pdf]

Appendix 3 - Data extraction and coding form

Multiple reports

How many reports are linked to this study?
If multiple reports, specify from what report data was extracted where appropriate and also specify relevant contradictions when encountered.

Bibliographic data

Repeat for each report:

- Authors (surnames and initials, in the order in which they appear in the report):
- Report title:
- Source (journal name, book title, website URL, etc., including all information needed for the full citation, i.e. volume, issue, page numbers, etc.):
- Year of publication or report, or date of communication:
- Publication status or type
    - Journal article
    - Book
    - Book chapter
    - Master or dissertation thesis
    - Conference paper, proceeding paper, meeting abstract
    - Working paper
    - Market research report
    - Government/agency research report
    - Unpublished manuscript
    - Other:
- DOI (if available):

Study design characteristics

Based on the quasi-experimental taxonomy features checklist (Reeves et al., 2017).

1. Was merit pay/the comparator allocated... (answer "yes" to more than 1 item, if applicable) (Yes/No/Possibly/Not applicable)
    a. to individuals?
    b. to clusters of individuals?
    c. clustered by the way it was provided?
2. Were outcome data available... (answer "yes" to only 1 item) (Yes/No/Possibly/Not applicable)
    a. after intervention/comparator only (same individuals)? If yes, specify (i.e. how long after announcement/allocation).
    b. after intervention/comparator only (not all same individuals)? If yes, specify (i.e. how long after announcement/allocation).
    c. before (once) and after intervention/comparator (same individuals)? If yes, specify (e.g. timing, duration between measurements).
    d. before (once) and after intervention/comparator (not all same individuals)? If yes, specify (e.g. timing, duration between measurements).
    e. Multiple times before and multiple times after intervention/comparator (same

individuals)? If yes, specify (e.g. year, duration between time points).
    f. Multiple times before and multiple times after intervention/comparator (not all same individuals)? If yes, specify (e.g. year, duration between time points).
3. Was the intervention effect estimated by... (answer “yes” to only one item) (Yes/No/Possibly/Not applicable)
    a. change over time (same individuals at different time points)?
    b. change over time (not all same individuals at different time points)?
    c. Difference between groups (of individuals or clusters receiving either intervention or comparator)? If yes, specify the number and nature of groups.
4. Did the researchers aim for control of confounding... (design or analysis) (answer "yes" to only one item) (Yes/No/Possibly/Not applicable)
    a. by using methods that control in principle for any confounding?
    b. by using methods that control in principle for time-invariant unobserved confounding?
    c. by using methods that control only for confounding by observed covariates?
5. Were groups of individuals or clusters formed by... (answer “yes” to more than 1 item, if applicable) (Yes/No/Possibly/Not applicable)
    a. Randomization?
    b. Quasi-randomization?
    c. Explicit rule for allocation based on a threshold for a variable measured on a continuous or ordinal scale or boundary (in conjunction with identifying the variable dimension, below)?
    d. Some other action of researchers?
    e. Time differences?
    f. Location differences?
    g. Management/HR decision-makers/practitioners?
    h. Participants' preferences?
    i. Policymaker?
    j. On the basis of outcome?
    k. Some other process? (specify)
6. Were the following features of the study carried out after the study was designed? (answer “yes” to more than 1 item, if applicable) (Yes/No/Possibly/Not applicable)
    a. Characterization of individuals/clusters before intervention?
    b. Actions/choices leading to an individual/cluster becoming a member of a group?
    c. Assessment of outcomes?
7. Were the following variables measured before the allocation of merit pay? (answer “yes” to more than 1 item, if applicable) (Yes/No/Possibly/Not applicable)
    a. Potential confounders
    b. Outcome variable(s)

Additional study characteristics

- Was the study conducted in one or more organizations?
- In what country or region was the study conducted?
- In what sector or industry was the study conducted?
- If applicable, did the pre-measurement of the outcome(s) take place before the implementation of the merit pay system was announced? (Yes/No/Uncertain)
- Was the merit pay system already in use before the start of the study? (Yes/No/Uncertain)
- Non-response/attrition (if applicable) - How many employees were invited to participate? How many employees participated (at each time point)? What information do the authors provide related to the risks for non-response bias/attrition?
- Funding sources for the study, if any.

Sample characteristics

- Age (range, mean, standard deviation)
- Gender (% per subgroup)
- Education level (% per subgroup)
- Ethnicity (%per subgroup)
- Seniority/organizational tenure (range, mean, standard deviation)
- Job level (% per subgroup)
- Promotion (% per subgroup)
- Sample size (per comparison group or per timepoint, if applicable)

Intervention characteristics

- What answer best describes the allocated merit increases?
  - Fixed amounts
  - Percentages of base salary
  - Unsure - contact author(s)
- What rating sources are consulted for performance appraisals on which the allocation of merit increases is based? Check all that apply.
  - Direct supervisor
  - Co-workers
  - Subordinates
  - Customers
  - Students
  - Other:
  - Unsure - contact author(s)
- What performance standards are rated in the performance appraisals on which the allocation of merit increases is based? Check all that apply.
  - Traits
  - Behavior/competencies
  - Goal attainment/Results
  - Other:
  - Unsure - contact author(s)
- What approach best describes the performance appraisal method?
  - Absolute comparison of the employee to a desired standard
  - Relative comparison of the employee against other employees through ranking
  - Forced ranking
  - Other:
  - Unsure - contact author(s)
- How often are performance appraisals conducted?
  - Quarterly
  - Semi-annually
  - Annually
  - Bi-annually
  - Other:
  - Unsure - contact author(s)
- How often are merit increases allocated?
  - Quarterly
  - Semi-annually
  - Annually
  - Bi-annually
  - Other:
  - Unsure - contact author(s)

- What factors, other than the performance appraisal, determine the size of the allocated merit increase? Check all that apply.
    - Available budgets, budgeting practices
    - Job level
    - Pay range attributed to the job
    - Position within the pay range (cf. compa-ratio)
    - Other:
    - Unsure - contact author(s)
- When are new merit increases allocated?
    - All employees at the same time. Specify month or quarter:
    - On the anniversary of the employee's employment contract
    - Other:
    - Unsure - contact author(s)
- Is the merit pay system applied to all employees in the organization or just a subgroup?
    - All
    - Subgroup. Specify:
    - Unsure - contact author(s)
- Are there any other aspects of the performance appraisal and merit pay system worth mentioning in relation to its context, configuration, or implementation?
    - Yes, specify:
    - No

Study results

Is motivation one of the outcomes measured in this study?

- No
- Yes, specify:
    - Motivation type (moderator): general motivation, behavioral intention, intrinsic, extrinsic, or other?
    - Source of the measure (i.e. citation)
    - Number of items
    - Response scale (e.g. 5-point Likert scale, 1 = totally disagree, 5 = totally agree)
    - Scale type: nominal, ordinal, interval, or ratio
    - Interpretation - Is a high score positive or negative?
    - Respondent: employee/self-rating; supervisor-rating; peer rating; other:
    - Reliability statistics and any additional information on validity
    - How was the data collected (e.g. paper-pencil survey, online survey, telephone interview, face-to-face interview)?
    - Who collected the data (e.g. researchers, HR, manager)?
    - Number of measurements: one after; before and after; before and several times (specify:) after one merit round; before and several times (specify:) after, each after successive merit rounds.
    - How long after the allocation of the merit increase was this outcome measured? Specify in months. If unsure, contact author(s).
    - Effects sizes: raw data, type of effect size (e.g. correlation, regression coëfficient), statistical method used, mean values, standard deviation or other measures for variability, n, p-value, etc.
    - For each effect size, if applicable, specify the number and what covariates were included in the model.
    - What theory do the authors invoke to explain the expected relationship between the intervention and this outcome?

Is effort one of the outcomes in this study?

- No
- Yes, specify:
  - Source of the measure (i.e. citation)
  - Number of items
  - Response scale (e.g. 5-point Likert scale, 1 = totally disagree, 5 = totally agree)
  - How is the scale score obtained? (Sum/average/weighted average/other:)
  - Scale type: nominal, ordinal, interval, or ratio
  - Interpretation - Is a high score positive or negative?
  - Respondent: employee/self-rating; supervisor-rating; peer rating; other:
  - Reliability statistics and any additional information on validity
  - How was the data collected (e.g. paper-pencil survey, online survey, telephone interview, face-to-face interview)?
  - Who collected the data (e.g. researchers, HR, manager)?
  - Number of measurements: one after; before and after; before and several times (specify:) after one merit round; before and several times (specify:) after, each after successive merit rounds.
  - How long after the allocation of the merit increase was this outcome measured? Specify in months. If unsure, contact author(s).
  - Effects sizes: raw data, type of effect size (e.g. correlation, regression coëfficient), statistical method used, mean values, standard deviation or other measures for variability, n, p-value, etc.
  - For each effect size, if applicable, specify the number and what covariates were included in the model.
  - What theory do the authors invoke to explain the expected relationship between the intervention and this outcome?

Is performance one of the outcomes in this study?

- No
- Yes, specify:
  - Performance type: general/task performance; contextual performance (specify: OCB or change-oriented behaviors)
  - Source of the measure (i.e. citation)
  - Number of items
  - Response scale (e.g. 5-point Likert scale, 1 = totally disagree, 5 = totally agree)
  - How is the scale score obtained? (Sum/average/weighted average/other:)
  - Scale type: nominal, ordinal, interval, or ratio
  - Interpretation - Is a high score positive or negative?
  - Respondent: employee/self-rating; supervisor-rating; peer rating; other:
  - Reliability statistics and any additional information on validity
  - Was performance data collected separately for the communicated purpose of research or did the authors rely on organizational records of performance ratings?
  - How was the data collected (e.g. paper-pencil survey, online survey, telephone interview, face-to-face interview, company records)?
  - Who collected the data (e.g. researchers, HR, manager)?
  - Number of measurements: one after; before and after; before and several times (specify:) after one merit round; before and several times (specify:) after, each after successive merit rounds.
  - How long after the allocation of the merit increase was this outcome measured? Specify in months. If unsure, contact author(s).
  - Effects sizes: raw data, type of effect size (e.g. correlation, regression coëfficient), statistical method used, mean values, standard deviation or other

    measures for variability, n, p-value, etc.
  - For each effect size, if applicable, specify the number and what covariates were included in the model.
  - What theory do the authors invoke to explain the expected relationship between the intervention and this outcome?

Does the study report the actual association between the performance appraisals and the size of the merit increases? Or can it be inferred?

- No - Contact author's
- Yes - Type of association measure, mean value, standard deviation or other measure for variability, n, p-value

Did the study measure the perceived association by employees between performance and pay?

- No
- Yes, specify:
  - Source of the scale (i.e. original reference)
  - Number of items
  - Response scale (e.g. 5-point Likert scale, 1 = totally disagree, 5 = totally agree)
  - How is the scale score obtained? (Sum/average/weighted average/other:)
  - Scale type: nominal, ordinal, interval, or ratio
  - Interpretation - Is a high score positive or negative?
  - Respondent: employee/self-rating; supervisor-rating; peer rating; other:
  - Reliability statistics and any additional information on validity
  - Raw data: mean, standard deviation, n

Risk of bias assessment

For assessing the risks of bias in each study per outcome of interest, we will use the ROBINS-I tool (Risk Of Bias In Non-randomized Studies - of Interventions) (Sterne et al., 2016). It considers 7 domains of bias, namely (1) bias due to confounding, (2) bias in the selection of participants into the studies, (3) bias in the classification of interventions, (4) bias due to deviations of intended interventions, (5) bias due to missing data, (6) bias in the measurement of outcomes, and (7) bias in the selection of the reported results. The tool itself, an empty template of the tool, and background information and detailed guidance for using the tool, can be retrieved at https://www.riskofbias.info/welcome/home/current-version-of-robins-i.
